# The therapeutic potential of zinc-nanoparticles against the cerebellar neurotoxicity induced by bacterial lipopolysaccharides in female rats and their pups

**DOI:** 10.1038/s41598-026-50012-4

**Published:** 2026-05-04

**Authors:** Abd El-Fattah B. M. El-Beltagy, Mai Eladad, Karoline Kamel, Hassan I. H. Elsayyad, Amany Attallah

**Affiliations:** 1 Zoology Department, Faculty of Science, Damanhour University, Damanhour, Egypt; 2 Zoology Department, Faculty of Science, Mansoura University, Mansoura, Egypt

**Keywords:** LPS, Gestation, Pups, Cerebellum, Oxidative stress, Apoptosis, Biochemistry, Cell biology, Neuroscience

## Abstract

Lipopolysaccharide (LPS) is an immunostimulatory endotoxin component of the cell wall of Gram-negative bacteria. LPS correlates with unfavorable developmental consequences, such as preterm birth, fetal demise, teratogenic effects, and intrauterine growth restriction. Zinc is essential for embryogenesis, fetal development, and lactation. This study sought to assess the efficacy of zinc nanoparticles (Zn-NPs) in alleviating cerebellar toxicity caused by LPS in female rats and their offspring. Treatment of LPS-administered female rats with Zn-NPs led to a significant restoration of oxidative stress levels, as indicated by a substantial reduction in malondialdehyde and a marked improvement in superoxide dismutase catalase activity, and Glutathione content in the cerebellar tissues. A significant enhancement was observed in the lipid profiles (total cholesterol, triglycerides, low-density lipoprotein, and high-density lipoprotein) and neurotransmitters (serotonin and dopamine). This was accompanied by significant restoration in the histological and ultrastructural architecture of the cerebellar cortex damaged by LPS. Furthermore, the treatment of LPS-administered mothers with Zn-NPs significantly mitigated the neurodegenerative alterations generated by LPS, as demonstrated by the regulation of synaptophysin, chromogranin, and neuron-specific enolase. Conclusion: Zinc nanoparticles could potentially have a therapeutic role by mitigating cerebellar neurotoxicity caused by bacterial LPS during gestation by reducing oxidative stress and apoptosis.

## Introduction

Neurotoxicity implies the direct or indirect impact of any potentially deleterious substance on the neurological system of individuals or animals^1,2^. Certain neurotoxins exert direct effects on brain cells^3^, whilst others modify metabolic processes that are significantly reliant on the neural system. Symptoms may manifest immediately upon exposure or may be delayed^2^. The nervous system elicits various functional responses to neurotoxic chemicals, resulting in a range of neurological disorders^1^.

Exposure to infectious pathogens during gestation is significantly associated with adverse neonatal outcomes, including harmful neurodevelopmental effects and enduring impairments such as cognitive dysfunction, learning disabilities, memory deficits, and altered social behavior that continue into adolescence and adulthood^4,5^. Lipopolysaccharide (LPS) is a deleterious element of the cell walls in Gram-negative bacteria and is extensively utilized to create a recognized model of bacterial infection. LPS is extensively utilized as a potent inflammatory agent to provoke neuro-inflammatory responses in several animal models^6–8^. In mice, early-life exposure to LPS stimulates the synthesis of pro-inflammatory cytokines in both peripheral tissues and the brain^9^. LPS injections induce acute responses that initiate systemic inflammation associated with numerous prevalent illness conditions throughout life. Included among these conditions are maternal immune activation^10^ and the etiology of cardiovascular diseases, chronic kidney diseases, autoimmune disorders, ovarian diseases, cancer, depression, and neurodegenerative disorders^11–13^.

In pregnant rodents, a pro-inflammatory response was detected in maternal and fetal plasma as well as in amniotic fluid following LPS administration^14,15^. This inflammation increases oxidative stress in both the mother and fetus^16,17^. Exposure to low doses of LPS during gestation leads to intrauterine growth restriction and metabolic dysregulation^18,19^. Exposure of pregnant rodents to LPS during the first trimester led to embryonic resorption and fetal demise^20,21^. Maternal exposure to LPS during the second trimester resulted in fetal demise and premature delivery^22^. Exposure to LPS in the third trimester resulted in fetal death, growth restriction, delayed skeletal development, premature labor, and testicular dysfunction in fetuses^23–25^.

LPS elevates intracellular reactive oxygen species (ROS) levels and promotes oxidative stress^26,27^. In the presence of LPS, the amount of glutathione peroxidase-4 (GPX4) diminishes, although the expression of antioxidant enzymes, including SOD and CAT, increases^27^. However, in the presence of elevated levels of reactive oxygen species (ROS), these enzymes are incapable of mitigating oxidative stress^29^. Prenatal exposure to LPS may result in structural damage and malfunction of hippocampus neurons and the cerebral cortex^30,31^. Prior findings indicated that maternal LPS exposure during mid to late gestation resulted in age-dependent deficits in neurobehavioral development, including spatial learning and memory, anxiety and exploratory behavior, as well as sensorimotor and species-typical behaviors in adult offspring^32,33^. Nevertheless, limited information exists regarding the impact of LPS exposure on the cerebellum of dams and their offspring.

Zinc is an essential trace element found in all tissues and bodily fluids. As a fundamental physiological component of the antioxidant defense system, it significantly contributes to cellular signaling^34,35^. Zinc regulates about 300 metalloenzymes and is essential for signal transmission, cellular proliferation, apoptosis, gene expression, and brain development in infants^36,37^. Zinc, as a component of several proteins, is crucial for multiple anabolic processes, protein synthesis, and nucleic acid metabolism^38^. Ensuring zinc homeostasis is essential for optimal brain function, since both zinc deficiency and excess can lead to brain injury and worsen neurological disorders^39,40^. Zinc is involved in both physiological and pathological processes inside the central nervous system^41–43^. The telencephalon, particularly the cortical region, hippocampus, and amygdala, serves as a primary storage location for zinc^44^. Zinc is essential to the antioxidant defense system. It serves as a cofactor for various antioxidant enzymes, including SOD, which transforms superoxide radicals into less reactive entities. Zinc aids in stabilizing cell membranes and safeguarding against lipid peroxidation^45^. Zinc significantly influences cognition, emotional stability, and memory^46^.

Zinc is crucial to proper embryogenesis during pregnancy^37^. The transmission of zinc to the fetus mostly relies on sufficient maternal zinc levels^47,48^. Consequently, premature pups are at significant risk of insufficiency owing to restricted zinc transfer from mother to fetus^49^. Zinc is sequestered in the liver as zinc-binding protein (metallothionein) to satisfy fetal requirements and to safeguard the fetus from zinc deficit during the initial postnatal phase^37,50^. Zinc is essential for cellular proliferation, differentiation, and the development of fetal organs during the early stages of pregnancy. Furthermore, its lack, particularly during late pregnancy, negatively impacts proper neuronal replication, migration, synaptogenesis, and gene expression^51^. Zinc shortage in pregnancy disrupts cell cycle progression, cell migration, intracellular signaling, and the normal operation of zinc-dependent enzymes, resulting in chromosomal and oxidative damage^44,52^.

Nanoparticles^53^, such as zinc oxide (ZnO), demonstrate potential in several applications, including medication administration and biomarker detection^54^. These nanoparticles can penetrate the brain, particularly the cerebellum, cortex, and olfactory bulbs, hence enhancing their medicine bioavailability^55–57^. Zinc nanoparticles^53^ are among the most prevalent nanoparticles utilized in diverse domains such as cosmetics, sunscreen, rubber composites, and food additives^53^. They exhibit significant antibacterial, anticancer, antidiabetic, and anti-inflammatory properties attributable to their antioxidant activity and essential functions in DNA replication, DNA repair, and cell division^58,59^. Furthermore, Zn NPs are crucial for preserving cell membrane integrity and regulating ROS metabolism by reducing lipid peroxidation and augmenting antioxidant enzyme activity^13,60^.

LPS exposure during pregnancy has been shown to cause maternal and fetal hypozincemia, leading to aberrant neurodevelopment and alterations in both the function and structure of the offspring’s brain^61–63^. The cause of harm remains ambiguous, however it may be partially attributed to zinc’s role as an essential trace mineral necessary for the proper functioning of numerous enzymes and transcription factors involved in brain development and neurogenesis^64^. This study seeks to assess the therapeutic efficacy of Zn-NPs in mitigating bacterial LPS-induced cerebellar toxicity in albino rat dams and their offspring.

## Material and methods

### Bacterial Lipopolysaccharides (LPS)

Lipopolysaccharide (LPS) (From the walls of Escherichia coli, serotype 055:B5 was purchased from Sigma–Aldrich Co. LLC, GmbH, Steinheim, Germany.

### Preparation of Zinc nanoparticle (Zn-NPs)

**Fig. 1:**
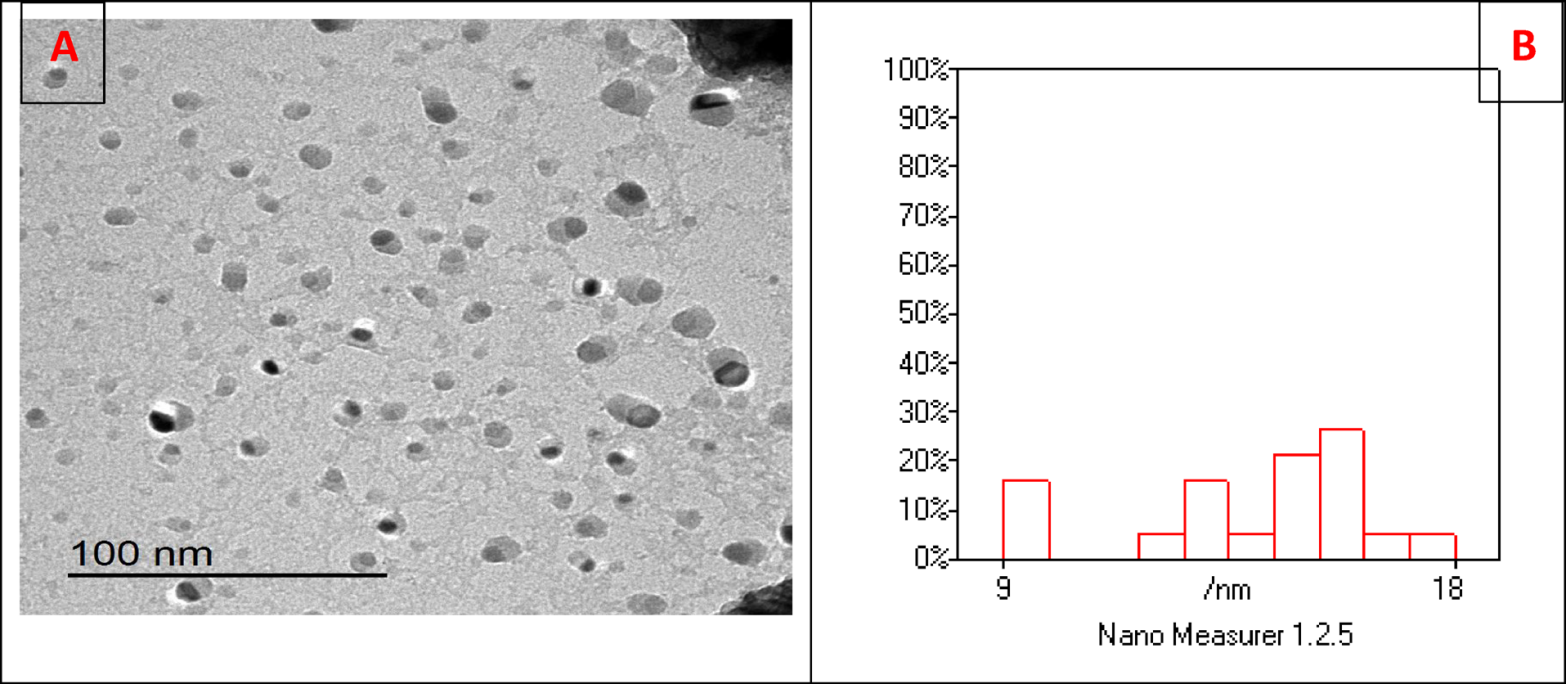
(**A**) The morphology of zinc nanoparticles by TEM showing nearly cubic or spherical particles. (**B**) Histogram shows that most particles cluster around 9 to 18 nm, with a good distribution.

Zn-NPs were commercially purchased from Alfa Nanomaterials Chemistry (Colin DrHolbrook, USA). The nanoparticles had a purity of 99.9%, an average particle size of 15 ± 5, and were provided as dry powder. For experimental use, Zn-nano-powder was suspended in deionized water at concentrations of 500 and 2000 μg L^−1^. To prevent agglomeration, the suspension was dispersed via ultrasonic vibration at 40 kHz for 30 min. A fresh suspension of Zn-NPs was prepared daily. To verify that the exposure concentrations align with the preset values, Zn concentrations in the exposed water were quantified using inductively coupled plasma mass spectrometry (ICP-MS) at 0, 12, and 24 h of exposure. The transmission electron microscope (TEM) (JEM-1011, JEOL, Japan) was employed to ascertain the morphology and dimensions of Zn-NPs. The measured particle size of the Zn-NPs closely aligned with the manufacturer’s specified cations, exhibiting almost cubic or spherical shapes compatible with nanoscale dimensions. Zeta potential and polydispersity index (PDI) tests were conducted on hydrated nanoparticles to evaluate the surface charge and particle size distribution (Malvern Panalytical, Malvern, UK). The majority of particles clustered between 9 and 18 nm, exhibiting a favorable distribution (Fig. 1).

### Experimental design

Thirty-two female albino rats, each weighing 180± g, were procured from the Holding Company for Biological Products and Vaccines (VACSERA, Cairo, Egypt). The rats were kept in wire-bottom cages inside an environment featuring a conventional 12-hour light-dark cycle, a temperature of 25 °C, and a relative humidity of 50%. They were provided with tap water and ad libitum-balanced food. Following a week of acclimatization, the animals were mated in designated mating cages (1 male: 2 females) nightly. Following pregnancy confirmation using vaginal smear and plug methods, the pregnant rats were categorized into four groups (8 per group) as outlined below:

- Group 1 (control): Pregnant rats were administered with standard food and purified water.
- Group 2: They received standard nourishment and were administered Zn-NPs (20 mg/kg body weight/day) by oral gavage from gestation day 15 (E15) until the conclusion of weaning (a total of 28 days)^13^.
- Group 3 (LPS): Pregnant rats received two intraperitoneal injections of LPS, each at a dosage of 150 µg/kg, on gestation days E6 and E9^25^.
- Group 4 (LPS & Zn-NPs): This group received treatment with LPS and Zn-NPs at identical dosages and timings as those administered to groups 2 and 3.

### Evaluation of body weight

The body weight of maternal rats was assessed on gestation day 16 (GD-16) and postnatal day 21 (PND-21)^13^. Additionally, for each group, the total number of pups was documented and weighed on postnatal days 1, 7, 14, and 21.

### Euthanasia and tissue collection

At postnatal days 7 (PND-7), three pups were decapitated while at 21 (PND-21), one dam from each litter (at PND-21 only) and one male and one female pup per litter (n=8 pups/sex/group per time point) were euthanized for tissue collection. PND-7 pups were used exclusively for histological evaluation of cerebellar development, while PND-21 animals (both dams and pups) were used for all analyses (biochemical, immunohistochemical, flow cytometric, and ultrastructural studies). Euthanasia was conducted via quick decapitation, without prior anesthesia, to preserve labile neurochemicals for future biochemical and flow cytometric investigation. This approach was essential to promptly cease metabolic activity and prevent distortion of the study of sensitive endpoints. The surgery was executed promptly by skilled staff to reduce potential discomfort. Blood from the trunk was taken in sterile glass tubes. Serum was isolated using centrifugation at 3000 ×*g* for 10 minutes at 4 °C and thereafter kept at − 80 °C. The brain was swiftly excised, and the cerebellum was meticulously dissected on a chilled plate. The cerebellum was sectioned sagittally. One hemisection was immersed in 10% neutral buffered formalin for 48 hours for further histological analysis. The contralateral hemisection was rapidly frozen in liquid nitrogen and preserved at − 80 °C for biochemical and flow cytometric evaluation.

### Serum biochemical analysis

Serum was used for neurotransmitter analysis to promote association with lipid profiles from identical samples, as circulating neurotransmitter levels have been demonstrated to reflect central nervous system activity during inflammatory circumstances^65^. At PND-21, blood samples were obtained from the tails of both the maternal rats and their offspring. The blood samples were separated using centrifugation at 3000 rpm for 10 minutes to separate serum and plasma, subsequently kept at −80°C for further biochemical investigation.

#### Determination of serotonin and dopamine levels

An enzyme-linked immunosorbent test^12^ was performed utilizing commercially available kits to quantify plasma dopamine **(**Cat # EA0020Ca.**,** MyBioSource,USA) and serotonin (Cat # E0263Ca., Abcam, UK).The technical procedures adhered to the manufacturer’s guidelines^66^.

#### Measurement of lipid profiles

The serum lipid profiles have been assessed using a colorimetric assay kit in accordance with the manufacturer’s guidelines (Spinreact, Spain). The lipid fractions comprise triglycerides (TG), total cholesterol (CHO), high-density lipoprotein (HDL), and low-density lipoprotein (LDL). The serum total cholesterol level was assessed using a total cholesterol kit obtained from Spinreact SAU Ctra. Santa Coloma, SPAIN (Cat. NO. BSIS11-E)^67^**.** The serum TG level was assessed using a triglycerides kit obtained from Spinreact S.A.U., Ctra. Santa Coloma, SPAIN (Cat. NO. MIBSIS49E)^68^. Serum HDL was quantified utilizing a kit acquired from Spinreact S.A.U., Ctra. Santa Coloma, Spain (Cat. No. BSIS12-E)^69^ Serum LDL was computed as per the equation: LDL-c = TC—(HDL + TG/5)^70^. The concentration of each lipid fraction in serum is denoted in mg/dl.

### Measurement of antioxidants and oxidative stress markers in the cerebellar tissues

Cerebellar tissues were weighed and homogenized in ice-cold phosphate-buffered saline (PBS, 50 mM, pH 7.4) containing 1 mM EDTA to prevent metal-catalyzed oxidation. Homogenization was performed using a glass-Teflon homogenizer at 4 °C with 10 strokes at 1000 rpm. The homogenates were centrifuged at 10,000 ×*g* for 15 minutes at 4 °C, and the supernatants were collected for immediate analysis of antioxidant parameters and oxidative stress markers. Protein concentration in the supernatants was determined by the Bradford method using bovine serum albumin as standard. The activities of antioxidant enzymes, including superoxide dismutase (SOD) and catalase (CAT), as well as the quantity of glutathione (GSH), were evaluated in the cerebellum tissue homogenate. The levels of SOD, CAT, and GSH were quantified using a colorimetric approach with commercial kits from Biodiagnostic, Cairo, Egypt. SOD activity was assessed by the enzyme’s capacity to impede the reduction of nitroblue tetrazolium (NBT) by superoxide^71^.The activity of the CAT enzyme was assessed using the method outlined by Aebi^72^. The glutathione (GSH) content was assessed using commercially available kits (Biodiagnostic, Egypt) in accordance with the manufacturer’s recommendations^73^. Oxidative stress indicators such as malondialdehyde (MDA) and nitric oxide (NO) were evaluated in cerebellar tissues. MDA was evaluated by measuring the concentration of thiobarbituric acid reactive substances (TBARS), which was utilized to quantify the degree of MDA produced due to membrane lipid peroxidation^74^. No concentration has been determined using the sandwich enzyme immunoassay^12^ technology (BIOSOURCE, Europe S.A., Belgium, Lot No. 051501/B; 060601).

### Histological investigation of cerebellum

Cerebella from mothers and their pups (at PND-7&PND-21) preserved in formalin were dehydrated using an escalating sequence of ethanol, cleaned with xylene, and embedded in paraffin. Longitudinal slices of the cerebellum, measuring 5-6 μm in thickness, were procured and stained with hematoxylin and eosin^75^. The acquired sections were examined using a bright field light microscope and subsequently photographed.

### Immunohistochemical labeling of chromogranin A, synaptophysin and neuron specific enolase (NSE)

Paraffin sections perfused cerebellum from control and experimental groups of mother rats and their pups at postnatal day 21 were utilized to assess the immunoreactivity of chromogranin A, synaptophysin, and NSE. The sections were de-waxed and treated for one hour at room temperature in 0.3% hydrogen peroxide in phosphate-buffered saline, pH 7.6 (PBS). The slides underwent three washes (10 minutes each) in the identical buffer to inhibit endogenous peroxidase activity. They were incubated for 16 hours at 4 °C in PBS supplemented with 2% normal goat serum (NGS) and 0.5% Triton X-100, followed by a wash in PBS at room temperature. Subsequently, overnight incubation was conducted at 4 °C with the primary monoclonal antibodies targeting chromogranin A (LK2H10 and PHES), synaptophysin (1:100 dilution, Dako), neurofilament (1:800, Novus Biologicals), and NSE (Zymed, Carlton Court, San Francisco). After the primary antibody incubation, the sections underwent a 10-minute treatment at room temperature with streptavidin peroxidase (cat. No. TP-125-HL; Thermo Fisher Scientific, Inc.) and biotinylated goat anti-polyvalent secondary antibody. The sections were washed with PBS between treatments. 3, 3′-diaminobenzidine (Thermo Fisher Scientific, Inc.) was employed to elucidate the antibody binding locations. The sections were examined with a light microscope (Olympus, Tokyo, Japan) at magnifications of x100 and x400, following a five-minute counterstaining with Mayer’s haematoxylin at room temperature. The staining intensity of antibodies in cerebellar tissues was evaluated using image analysis software (version 1.52; National Institutes of Health)^76^.

### Transmission electron microscope investigation (TEM)

The ultrastructural analysis by TEM focused primarily on the cerebellar cortex cells of the mother rat and her offspring at postnatal day 21. Following the dissection of the chosen animals, tiny fragments of 1mm^2^ of cerebellar tissue were immersed in newly made 2.5% cold glutaraldehyde as a fixative for approximately five hours. Samples were subsequently rinsed in two aliquots of cold phosphate buffer, pH 7.3, for one hour^77^. The specimens underwent post-fixation for 1-2 hours in buffered 1% osmium tetroxide^78^, followed by buffer washing, dehydration in a graded series of cold ethyl alcohol, clearing in propylene oxide, and mounting in epoxy resin. Semi-thin sections with a thickness of 0.7 μm were prepared using glass blades on the 6000 MT RMC ultra-microtome. The sections were affixed to glass slides and dyed with 0.25% toluidine blue^79^. Thin sections were excised from a designated region (cerebellar cortex cells) for electron microscopy preparations, thereafter, placed on copper grids and stained with lead citrate and uranyl acetate as per Reynolds et al.^80^. The sections were subsequently analyzed and photographed using a JEOL 1200 EX11 transmission electron microscope in the EM Unit at Mansoura University.

### Flow cytometry detection of P53, tumor necrosis factor-α (TNF-α), and annexin-v in cerebellar tissues

Flow cytometric analysis was conducted on a FAC Scan (Becton Dickinson) with standard parameters: fluorescence 1 (FL1), 4 decades (logarithmic), detector voltage 648 V, logarithmic amplifier, compensation 1.1%; fluorescence 2 (FL2), 4 decades (logarithmic), detector voltage 496 V, logarithmic amplifier, compensation 22.8%. Data analysis was conducted utilizing software lysis (Becton Dickinson).

#### P53

Flow cytometric measurement of p53 expression was conducted utilizing a Coulter EPICS XL apparatus and EXPO 32 software (Beckman Coulter, Miami, FL, USA) as outlined by Newell et al.^81^. The cerebellar tissue cells were subjected to bulk lysis using buffered ammonium chloride, incubated with P53 antibody (PE-labeled clone 6C8; Pharmingen, San Diego, CA, USA) or the provided isotype control as per the supplier’s instructions for 20 minutes in darkness, then washed and re-suspended in phosphate-buffered saline containing 1% paraformaldehyde before analysis.

#### TNF-α

The cerebellum tissue samples were examined using a four-color flow cytometer (FACS Calibur, BD) with CELLQUEST software (BD). A minimum of 50,000 CD4+ T cells were obtained for each sample. Cells were gated by FSC vs SSC to isolate lymphocytes, followed by SSC versus CD4+ to separate CD4+ T cells. The exclusion channel (CD33/CD62P APC) was employed to exclude monocytes and activated platelets as detailed by Nomura et al.^82^. Dot plots illustrated events categorized as cytokine+ versus CD69+ T cells. The percentage of CD4+ T lymphocytes generating TNF-α was assessed in all groups.

#### Flow cytometry evaluation of annexin V in cerebellar tissues

Cerebellar cell apoptosis was evaluated via flow cytometry employing an annexin V FITC/PI labeling kit (Pharmingen, Becton Dickinson Co., San Diego, CA, USA). Forty-eight hours post-transfection, the collected cells were washed twice with PBS (sodium chloride NaCl 40.0 g, potassium chloride KCl 1.0 g, potassium dihydrogen phosphate anhydrous KH2PO4 1.0 g, disodium hydrogen phosphate anhydrous Na2HPO4 4.6 g, and distilled water to a final volume of 51 mL; 4 °C). The cells were re-suspended in the binding buffer (10 mM HEPES/NaOH pH 7.4, 140 mM NaCl, 2.5 mM CaCl2) and stained with fluorescein isothiocyanate-conjugated annexin V (annexin V-FITC)^83^.

### Statistical analysis

IBM SPSS software version 20.0 was used to analyze the data when it was uploaded to the computer (IBM Corp., Armonk, NY). The Shapiro-Wilk test was used to check continuous data. Data are expressed as mean ± standard deviation (SD) to reflect the biological variability within each experimental group. The four study groups were compared using the one-way ANOVA test, followed by pairwise comparison using the Post Hoc test (Tukey). At the 5% level, the findings were considered significant.

## Results

### Characterization of zinc nanoparticles:

Transmission electron microscopy (TEM) analysis revealed that the Zn-NPs were predominantly spherical or cubic in shape with particle sizes ranging from ca. 9 to 18 nm. Dynamic light scattering (DLS) measurements showed a zeta potential of -18.5 mV and a polydispersity index (PDI) of 0.21, indicating good colloidal stability and relatively uniform size distribution. These characteristics confirm the nanoscale dimensions and suitability of the particles for the experimental applications.

### Changes in the body weights of mother’s rats and their offspring

**Fig. 2:**
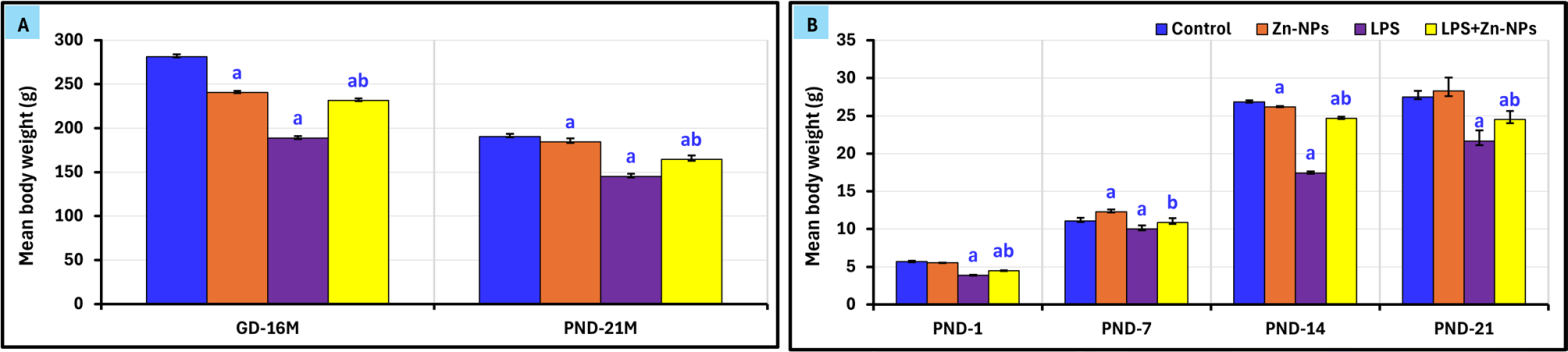
The mean body weight (g) of mothers’ rats at gestation day16 and PND-21(Panel **A**), and their offspring PND1, 7, 14, and 21(Panel **B**). ^*a*^* Significant with Control, *^*b*^* Significant between LPS and LPS* + *Zn-NPs group,* statistically significant at *P* ≤ 0.05. 5

The mean body weights of mother rats (GD-16 and at PND-21) and their pups at PND-21 are illustrated in Figure 2.

#### In mother rats

At GD-16, the average body weights of both Zn-NPs (240.5 g±0.76; *P*<0.05) and LPS-treated mother rats (188.6 g±0.9; *P*<0.05) were considerably reduced compared to the control group (281.4 g±0.95). Conversely, following the treatment of LPS-administered maternal rats with Zn-NPs, the average body weight exhibited a considerable rise compared to the LPS-only group (231.4±0.87; *P*<0.05), however remained significantly lower than that of the control group.

At PND-21, the average body weights of Zn-NPs-treated maternal rats (184.5 g±1.54) were significantly unchanged compared to the control group (190.6±1.11; *P*>0.05). The body weight of LPS-administered rats was considerably lower (145.13 g+1.14; *P*<0.05) compared to the control group. After treating LPS-administered maternal rats with Zn-NPs, their body weight dramatically increased compared to the LPS-administered group (164.5 g±1.73), however remained notably higher than the control group (*P*<0.05) (Figure2A).

#### In rat offspring

Offspring body weight was evaluated at postnatal days 1, 7, 14, and 21. The findings indicated that the average body weight of offspring at PND-1, 7, 14, and 21, maternally supplemented with Zn-NPs, exhibited no significant difference compared to the control (*P*˃0.05). Conversely, the average body weight of all pups subjected to maternal LPS induction was considerably lower (*P*<0.05) compared to the control group. All offspring maternally induced with LPS and subsequently treated with Zn-NPs exhibited a statistically significant increase (*P*<0.05) in body weight compared to their matching LPS-offspring; nevertheless, their weight was significantly lower (*P*<0.01) at PND-1 and PND-14 in comparison to the control group (Figure 2B). Indeed, LPS successfully induced significant growth restriction in both mothers and offspring while LPS+Zn-NPs Significantly better than LPS, but still significantly lower than Control.

### Changes in the levels of serum dopamine (DA) and serotonin (5-hydroxytryptamine or 5-HT)

**Fig. 3:**
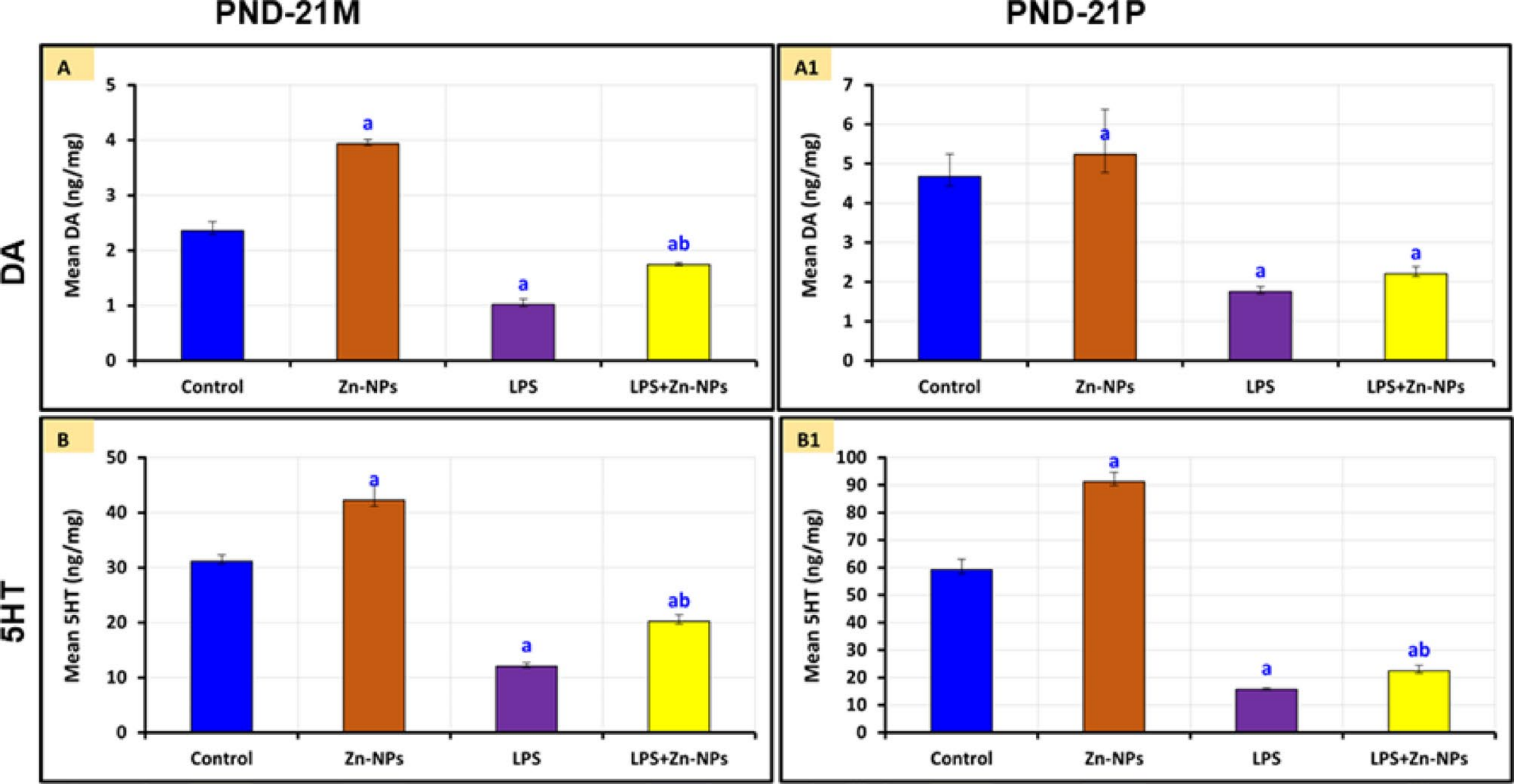
The mean levels of serum (DA) and (5-HT) among the control and experimental groups of mother rats (PND-21 M) and their pups at PND 21(PND-21P). ^*a*^* Significant with Control, *^*b*^* Significant between LPS and LPS* + *Zn-NPs group,* statistically significant at *P* ≤ 0.05.

In female rats administered LPS and their progeny, the levels of dopamine (DA) and serotonin^84^ were dramatically reduced (*P*<0.05) compared to the control group. In contrast, following treatment with Zn-NPs, the concentrations of DA and 5-HT were markedly increased (*P*<0.05) in both mothers and their progeny, although they remained significantly lower (*P*<0.05) than the control (Figure 3). Importantly, the LPS+Zn-NPs group showed significantly higher DA and 5-HT levels compared to the LPS-alone group (*P*<0.05) but remained significantly lower than both the control and Zn-NPs-alone groups (*P*<0.05). No significant differences were observed between the control and Zn-NPs-alone groups for any parameter."

### Changes in lipid profiles

**Fig. 4:**
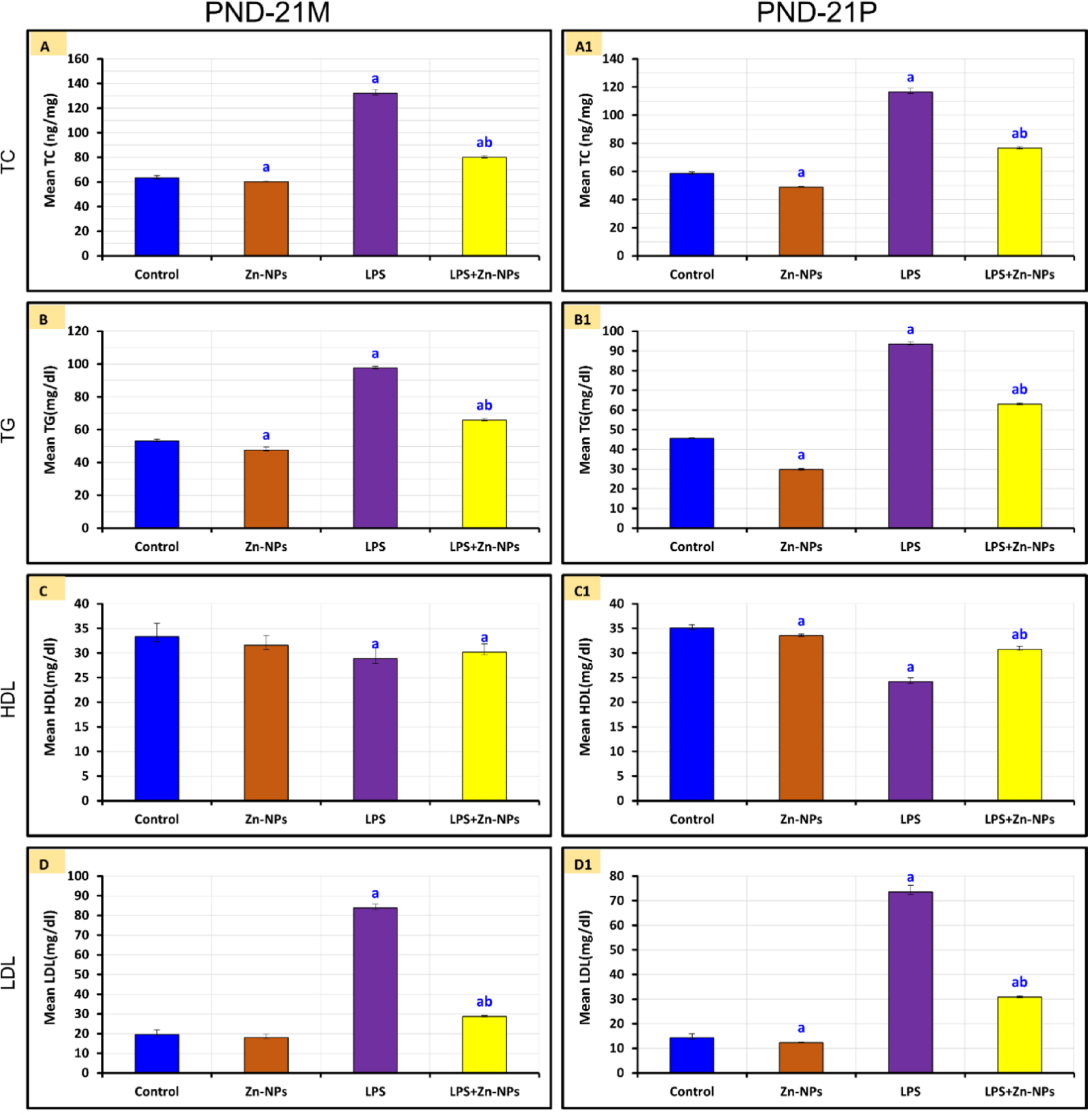
The mean level of serum TC, TG, HDL, LDL, among the control and experimental groups of mother rats (PND-21 M) and their pups at PND 21(PND-21P). ^*a*^* Significant with Control, *^*b*^* Significant between LPS and LPS* + *Zn-NPs group, statistically significant at P* ≤ *0.05.*

In female rats administered LPS and their PND 21 progeny, serum levels of TG, total cholesterol (TC), and low-density lipoprotein (LDL) were significantly elevated, whereas HDL levels were dramatically reduced (*P*<0.05) compared to the control group. Conversely, following treatment with Zn-NPs, a notable reduction in the levels of TG, TC, and LDL (*P*<0.05) was observed, alongside an increase in HDL levels (*P*>0.05) as compared to the LPS-administered group; nevertheless, these levels did not normalize to those of the control group (*P*<0.05) (Figure 4).

### Changes in antioxidants

**Fig. 5:**
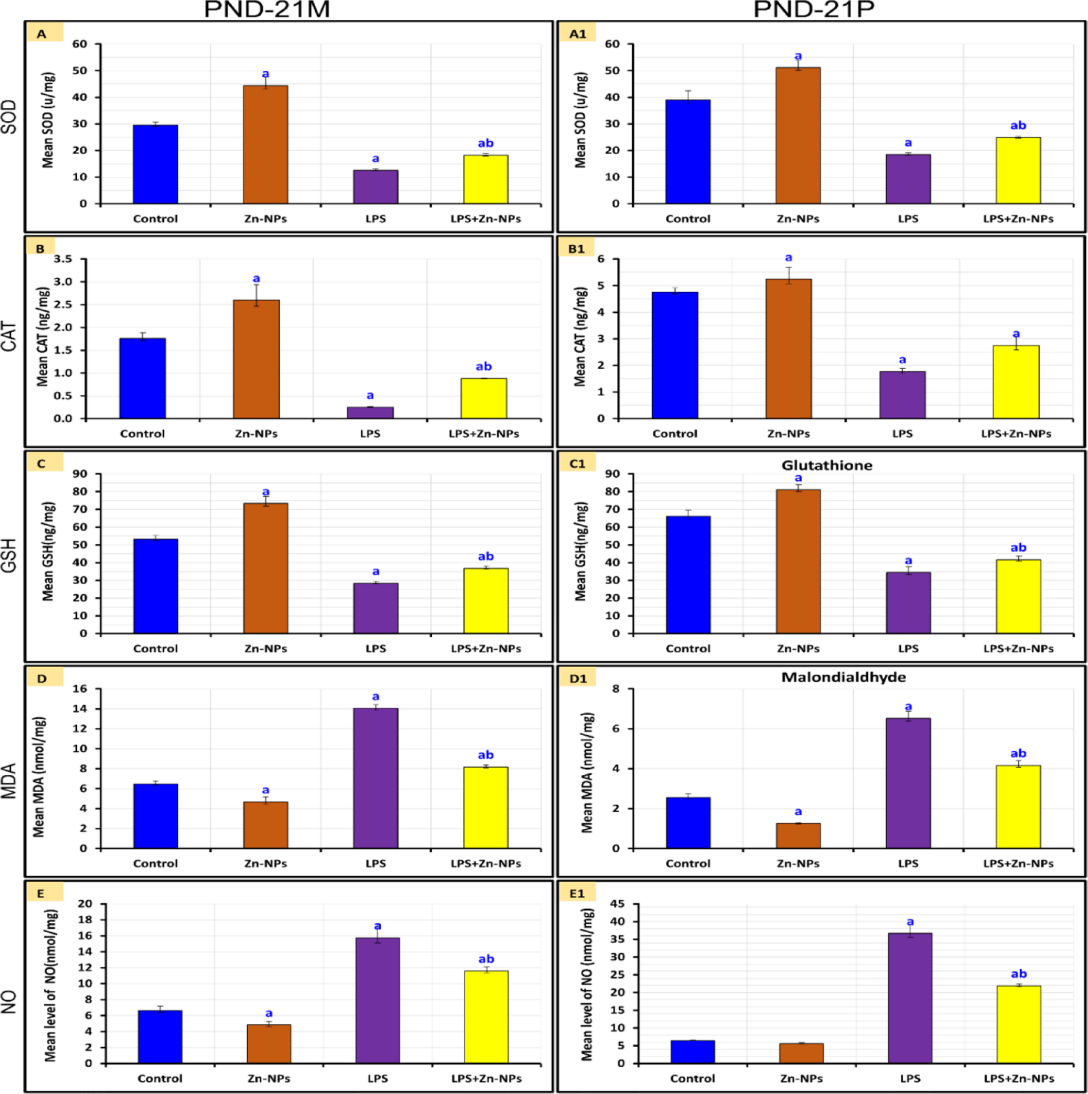
CAT and SOD activity, GSH content, and MDA and NO levels in the cerebellum of mothers rats (PND-21 M) and their pups (PND-21). ^*a*^* Significant with Control, *^*b*^* Significant between LPS and LPS* + *Zn-NPs group,* statistically significant at *P* ≤ 0.05.

Figure 5 illustrates that the cerebellar activities of CAT and SOD, along with GSH content, were significantly elevated (*P*<0.05), whereas MDA and Nitric oxide (NO) levels were dramatically reduced (*P*<0.05) in the Zn-NPs group of maternal rats and their offspring compared to the control group. In female rats and their pups given with LPS, the activities of CAT and SOD, as well as GSH content, were dramatically reduced (*P*<0.05), but the levels of MDA and NO were significantly increased (P<0.05) compared to the control group. In LPS-administered maternal rats supplemented with Zn-NPs and their offspring, the activities of CAT and SOD, as well as GSH levels, were significantly elevated (*P*<0.05), while MDA and NO levels were substantially reduced (*P*<0.05) compared to the LPS group alone; however, all parameters remained significantly altered (*P*<0.05) in relation to the control group

### Histopathological findings in the cerebellar cortex

#### In mother rats

**Fig. 6:**
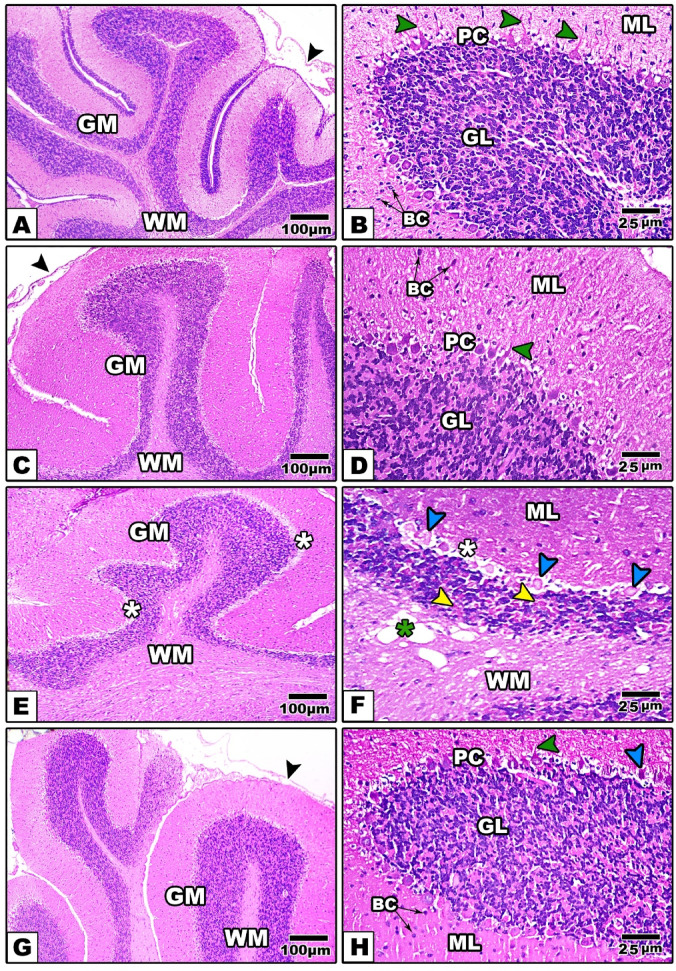
Images from the cerebellum histological sections of the control and experimental groups of mother rats (A, B: the control, C, D: Zn-NPs, E, F: LPS, and G, H: LPS & Zn-NPs group). In control and Zn-NPs groups, the cerebellar cortex shows well organized layers. In LPS-administered mother rats, the cerebellar cortex displays pyknotic Purkinje cells (blue arrow heads), atrophied granular cells (yellow arrow heads), remarkable separation between granular and Purkinje cell layer (white asterisk), scattered vacuoles in white matter (green arrow heads). In LPS + Zn-NPs-treated group, the cerebellar histoarchitecture illustrates remarkable amelioration that tend to be more or less as control (Stain: H&E, Scale bar: 100 µm in left-handed side images and 25 µm in right-handed images).Abbreviations: BC: Basket Cell; D: Dendrite, PC: Purkinje cell; GC: Granular Cells; PM: Pia Matter; ML: Molecular Layer; GL: Granular Layer; WM: White Matter. Scale bar: 100 μm in left-hand side images (original magnification × 100) and 25 μm in right-hand images (original magnification × 400)."

In both the control and Zn-NPs-treated maternal rats (Figure 6A-D), the cerebellar sections exhibited regular and abundant folia with proper differentiation into a broad outer cerebellar cortex^85^ covered by a thin layer of pia mater and a relatively narrower medulla (white matter). The cerebellar cortex exhibited three distinct strata: the outer molecular layer^61^, the inner granular layer (GL), and the intermediate Purkinje cell layer (PL). The ML exhibited sparse stellate and basket cells, distinguished by their typical basophilic cytoplasm. Both stellate and basket cells establish connections on the dendrites of Purkinje cells. This layer comprises the flattened dendritic trees of Purkinje cells and the extensive array of parallel fibers from the granular layer that intersect the Purkinje cell dendritic trees perpendicularly. The sizable, spherical somas of Purkinje cells are densely arranged in a monolayer of the cerebellar cortex, referred to as the Purkinje layer (PL). Purkinje cells are distinguished by their centrally positioned nuclei and acidophilic cytoplasm. The dendrites of Purkinje cells extend into the molecular layer, whereas their cell bodies reside in the granular layer. The granular layer, the third layer of gray matter, has around 10 rows of distinctly defined, darkly pigmented small nerve cells, characterized by tightly packed rounded and oval cells of varying sizes that impart a gritty appearance. The white matter is situated beneath the granular layer, which is devoid of cellular structures.

In LPS-administered mother rats, the ML layer of cerebellar cortex appeared histologically unchanged, while most of Purkinje cells are lost and some of which appeared with pyknotic nuclei. The granular layer showed relatively thinner thickness if compared with control, with remarkable atrophy and faintly stained granular cells. Additionally, several vacuoles were noticed in the white matter (Figure 6 E, F).

Supplementation of Zn-NPs to LPS-administered female rats from GD15 until weaning was successfully attenuated the cerebellar histopathological signs caused by LPS, whereas most of Purkinje cells appeared with their normal characteristic features as the control. Additionally, the granular layer appeared with normal thickness and scattered deeply stained granular cells (Figure 6 G, H).

#### In offspring

**Fig. 7:**
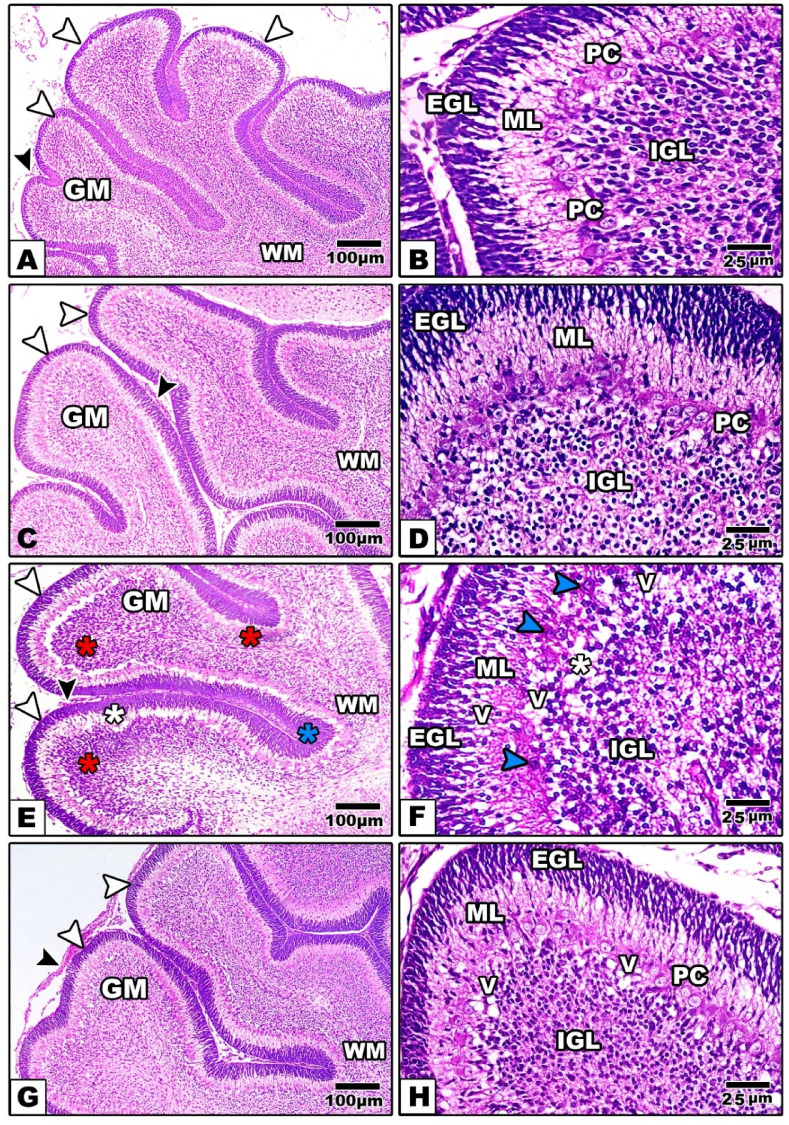
Images from the cerebellum histological sections of the control and experimental groups of offspring at PND-7 (A, B: the control, C, D: Zn-NPs, E, F: LPS, and G, H: LPS & Zn-NPs group). In control and Zn-NPs groups of offspring, the cerebellar cortex showing well organized four layers. In maternally LPS-treated offspring, the cerebellar cortex layers appear disorganized with pyknotic Purkinje cells (blue arrowhead) and several vacuolated cells (V). LPS + Zn-NPs-treated groups showing remarkable improvement in the histological feature of cerebellar cortex (Stain: H&E, Scale bar: 100 µm in left-handed side images and 25 µm in right-handed images). Abbreviations: GM: Gray Matter; WM: White Matter; EGL: external granular layer, IGL: internal granular layer; ML: Molecular Layer; PC: Purkinje cell; V: Vacuole. Scale bar: 100 μm in left-hand side images (original magnification × 100) and 25 μm in right-hand images (original magnification × 400)."

*At PND-7* In control and Zn-NPs maternally treated offspring at postnatal day 7, the cerebellar cortex exhibited four distinct cellular layers, a defining histological hallmark of the cerebellum at this developmental stage in rats. The layers comprised the exterior granular layer^86^, middle molecular layer^61^, Purkinje cell layer (PL), and internal granular layer (IGL) (Figure 7A–D).

On PND-7, the pups produced by maternal LPS exhibited a fragmented pia mater in the cerebellar cortex, significant cytoplasmic vacuolation, reduced granular cell density, and seemingly pyknotic Purkinje cells (Figure 7E, F). Nonetheless, the cerebellar cortex of offspring treated with LPS and Zn-NPs exhibited distinct delineation of cerebellar cortex layers, characterized by a well-distributed arrangement of granular and Purkinje cells, while a few vacuolated cells remained present in certain areas of the sections (Figure 7G, H).

**Fig. 8:**
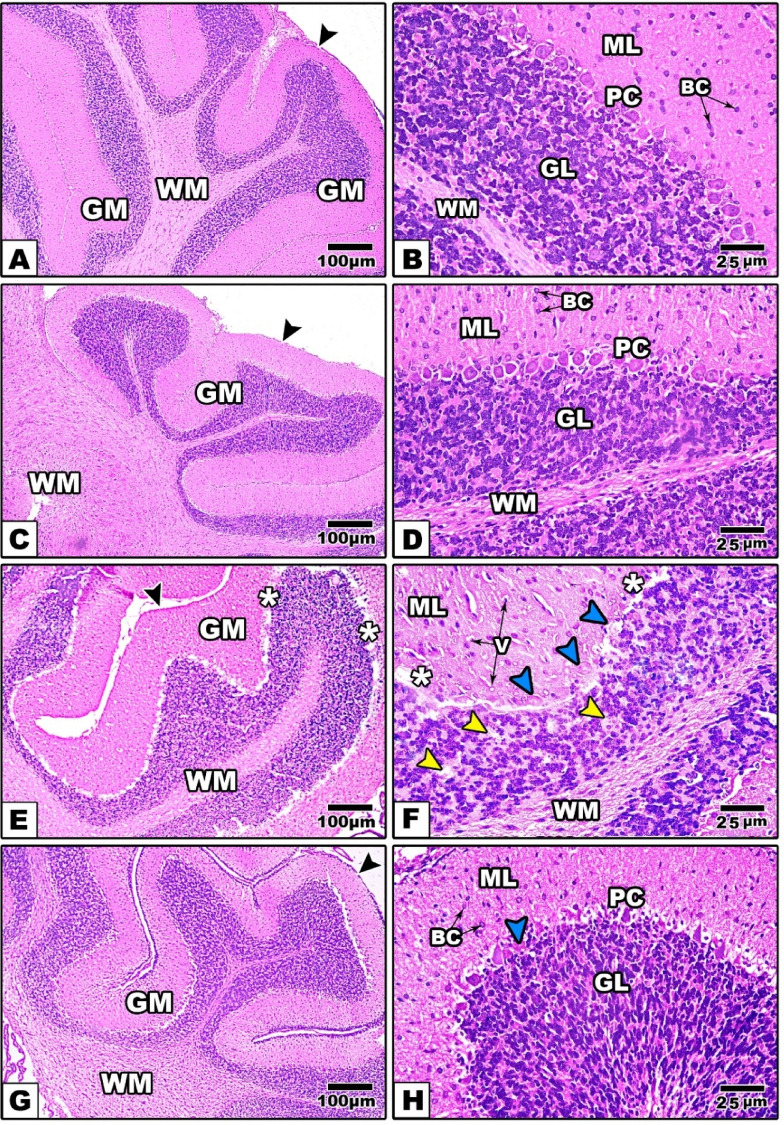
Photomicrographs of histological sections from the cerebellum of the different studied groups of offspring at PND-21 (A, B: the control, C, D: Zn-NPs, E, F: LPS, and G, H: LPS & Zn-NPs group). In control and Zn-NPs groups of offspring, the cerebellar cortex shows well organized layers (the layers appear thicker with condensed cells than those of mothers). In maternally LPS-treated offspring, the cerebellar cortex displays pyknotic Purkinje cells (blue arrowhead) and granular cells (yellow arrow heads) with remarkable separation between granular and Purkinje cell layer (white asterisk), and scattered vacuoles in molecular layer (V). Also, the granular layer appears relatively thinner than that of the control. LPS + Zn-NPs-treated group showing remarkable improvement in the histological feature of cerebellar cortex (Stain: H&E, Scale bar: 100 µm in left-handed side images and 25 µm in right-handed images). Abbreviations: BC: Basket Cell; D: Dendrite, PC: Purkinje Cells; GC: Granular Cells; PM: Pia Matter; ML: Molecular Layer; GL: Granular Layer; WM: White Matter. Scale bar: 100 μm in left-hand side images (original magnification × 100) and 25 μm in right-hand images (original magnification × 400)."

*At PND-21*, At PND-21 the cerebellar cortex from the control and Zn-NPs supplemented offspring appeared well organized and differentiated into the three ordinary layers; ML, GL, and PL as in adult rats with the exception that Purkinje cells and granular cells appeared condensed more than those of the adults (Figure 8A–D).

At 21-day old offspring maternally induced with LPS, the cerebellar cortex showed remarkable cell pyknosis especially in the granular and Purkinje cell layers. Also, several vacuoles were noticed in the granular and molecular layers (Figure 8E, F). After supplementation of Zn-NPs to LPS-treated mother’s rats, this was associated with remarkable restoration of cerebellar histopathological changes induced by LPS in their offspring (Figure 8G, H).

## Ultrastructural observations in the cerebellar cortex by TEM

In this study, the TEM investigations of the cerebellar cortex were focused on the three main cells of cerebellar cortex^87^, Purkinje (PC) and Basket cells (BC) (in both mother’s rats and their pups at PND-21.

**Fig. 9:**
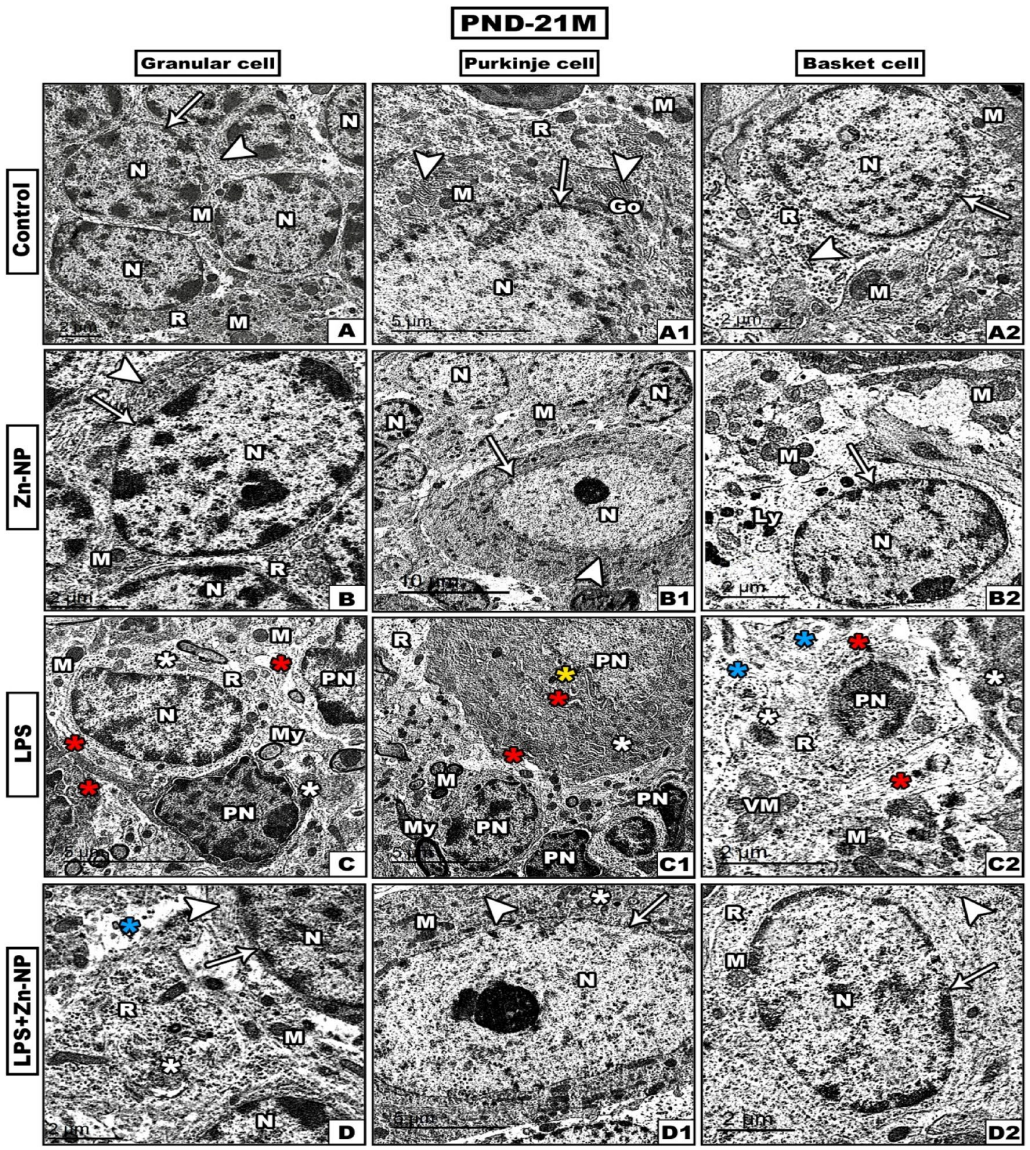
TEM through the cerebellar cortex cells (granular, Purkinje and basket cells) from the mothers’ rats (PND-21 M) among the control and experimental groups (A- A2: control, B-B2: Zn-NPs, C-C2: LPS, and D-D2: LPS + Zn-NPs). In control and Zn-NPs groups, the cells display normal subcellular architecture like intact nuclear membrane (arrows), rough endoplasmic reticulum (arrow heads). In LPS-administered female rats, the cerebellar cells showed intensive ultrastructure damage including pyknotic nuclei, irregular nuclear membrane (yellow asterisks), dilated rough endoplasmic reticulum (red asterisks), atrophied mitochondria (white asterisks), and vacuolated cytoplasm (blue asterisks). In LPS and Zn-NPs group, the cells showed an obvious amelioration to some extent in their fine structure. Abbreviations: Nucleus (N), Mitochondria (M), Ribosomes ®, Golgi apparatus (Go), Lysosomes (Ly), Myelin fibers (My), Pyknotic nucleus (PN) and Vacuolated mitochondria (VM).

**Fig. 10:**
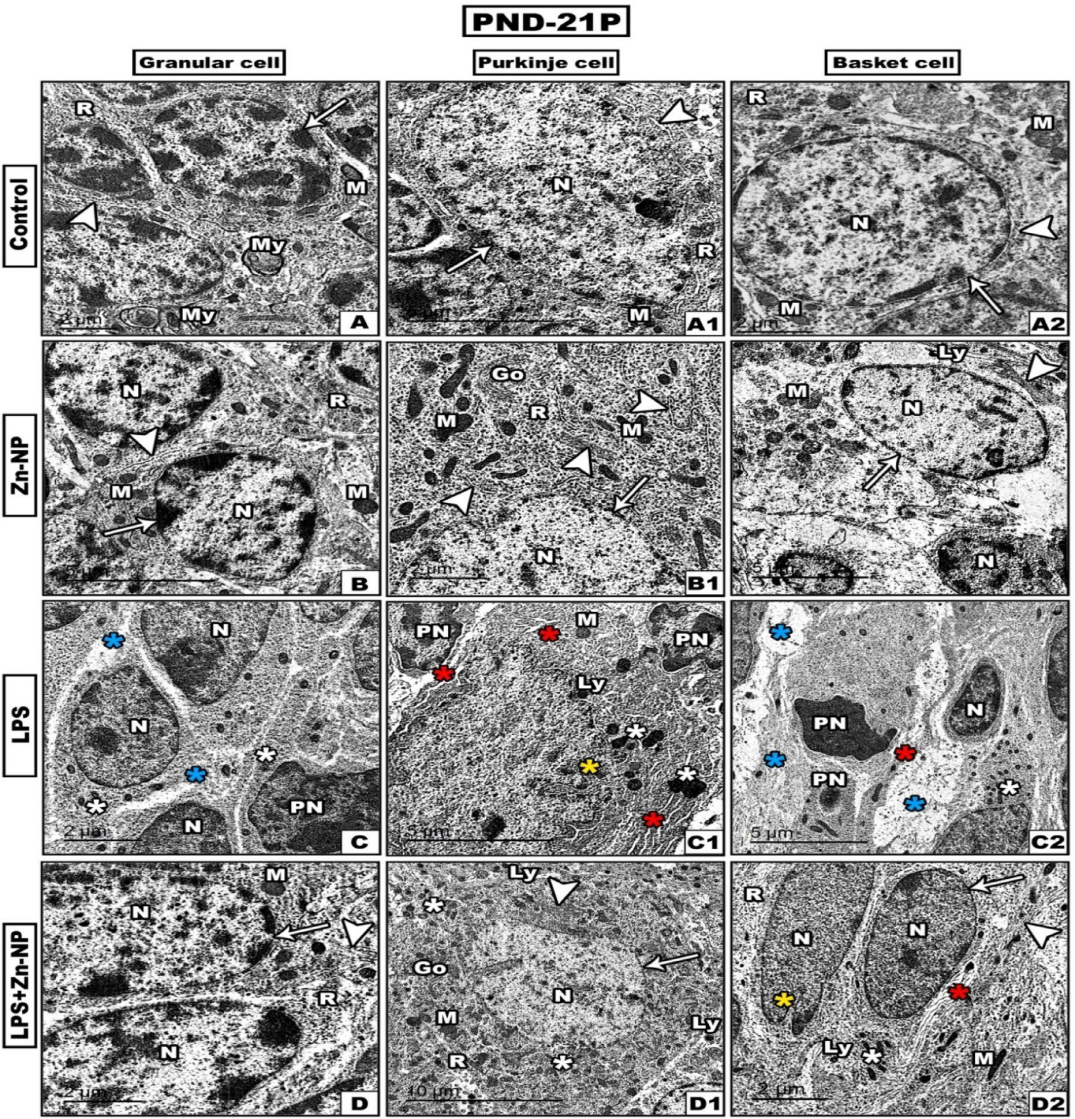
TEM through the cerebellar cortex cells (granular, Purkinje and basket cells) of the 21^st^ day old rats pup (PND-21P) among the different studied groups (A- A2: control, B-B2: Zn-NPs, C-C2: LPS, and D-D2: LPS + Zn-NPs). In control and Zn-NPs groups of pups, the cells showing normal subcellular architecture like intact nuclear membrane (arrows), rough endoplasmic reticulum (arrow heads). In LPS-induced pups, the cells showed intensive ultrastructure damage including Pyknotic nuclei (PN), irregular nuclear membrane (yellow asterisks), dilated rough endoplasmic reticulum (red asterisks), atrophied mitochondria (white asterisks), and vacuolated cytoplasm (blue asterisks). In LPS + Zn-NPS group of pups, the cerebellar cells reveal remarkable improvement in their fine structure which appears like the control. Abbreviations: Nucleus (N), Mitochondria (M), Ribosomes ®, Golgi apparatus (Go), Lysosomes (Ly), Myelin fibers (My), and Pyknotic nucleus (PN).

Transmission electron microscopy (TEM) analysis of the cerebellar cortex sections of the control and Zn-NPs supplemented dams (Figure 9A, B), and their 21-days old rats (Figure 10A, B) revealed that GCs contain central nucleus rich with heterochromatin and euchromatin, centrally located nucleolus and intact nuclear membrane. Moreover, the cytoplasm showed many dispersed mitochondria, ribosomes and the filaments of the endoplasmic reticulum. GCs dendrites appeared surrounded by deeply stained myelin sheath. The cerebellar PCs of mother rats (Figure 9A1, B1) and their offspring (Figure 10A1, B1) appeared large, uniform which had oval euchromatin, well-defined nucleolus and regular nuclear membrane. The cytoplasm showed numerous mitochondria with intact membranes and regular cristae, Golgi apparatus, free ribosomes and numerous well-developed rough endoplasmic reticula. Moreover, the cerebellar basket cells of (BCs) of mothers rats (Figure 9A2, B2) and their pups (Figure 10A2, B2) seemed typically organized as indicated by presence of regular nucleus, rough endoplasmic reticulum, numerous mitochondria, free ribosomes and lysosomes.

In LPS-injected dams (Figure 9C) and their pups (Figure 10C) the GCs revealed multiple pyknotic hyperchromatic nuclei (PN), atrophied mitochondria, dilated and damaged rough endoplasmic reticulum (RER). Moreover, the PCs of LPS-administered mothers (Figure 9C1) and their 21-days old offspring (Figure 10C1) showed dilated rough endoplasmic reticulum, atrophied or lysed mitochondria, and pyknotic nuclei with obvious irregular nuclear membrane. Regarding, the BCs in mother rats (Figure 9C2) and their pups (Figure 10C2) revealed pyknotic nucleus, little cytoplasmic organelles and dilated rough endoplasmic reticulum, vacuolated and atrophied mitochondria all-over the cell. Furthermore, the cerebellar cortex cells in LPS-administered mother rats (Figure 9D–D2) and their 21-days old rats (Figure 10D-D2) post-supplemented with Zn-NPs showed remarkable improvement in the fine structure of granular cells, Purkinje cells and basket cells as indicated by reconstruction of cell membranes and well homogenized cytoplasm and cell organelles.

## Immunohistochemical observations in the cerebellar cortex

### Chromogranin A immunohistochemical activity

**Fig. 11:**
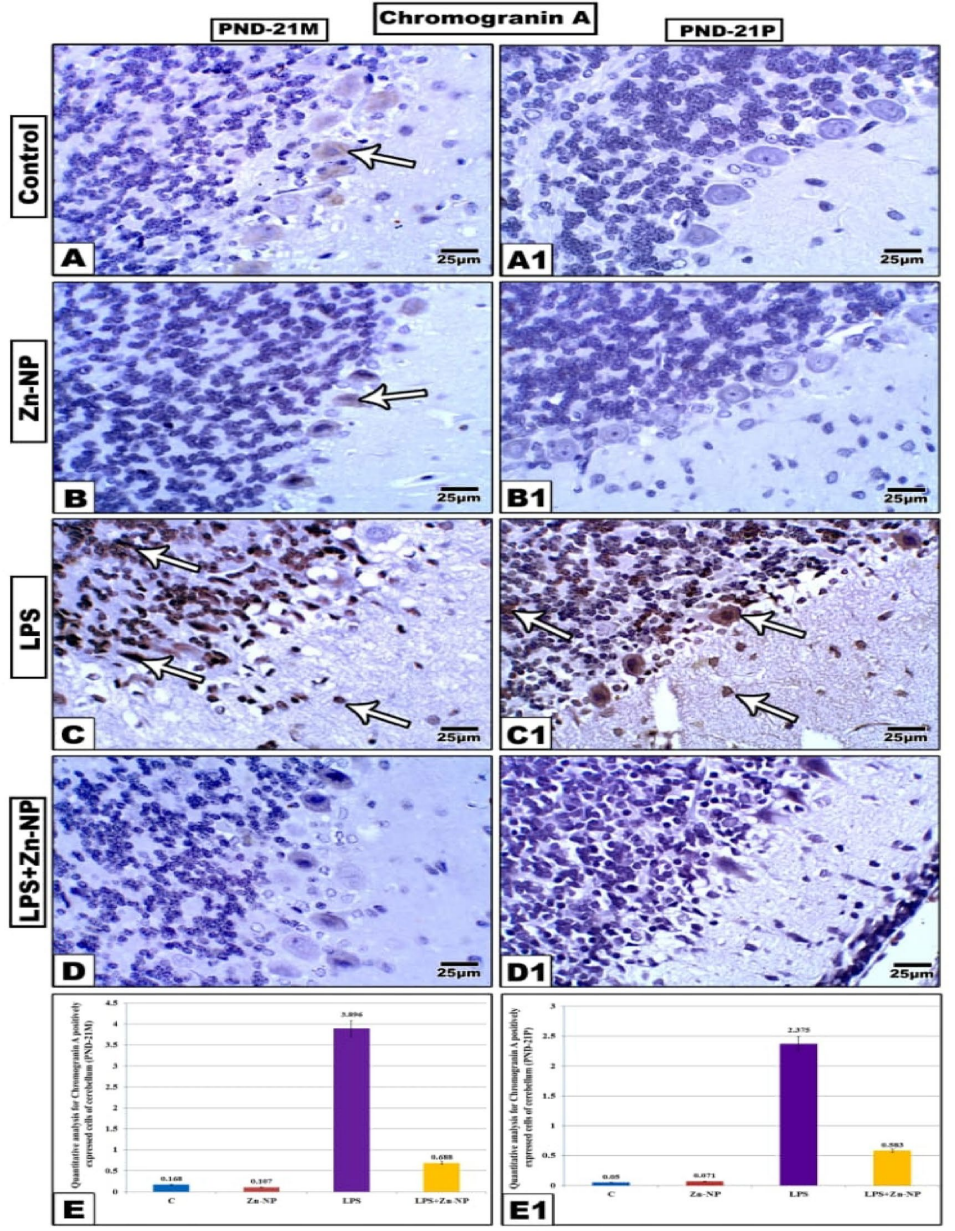
Expression of chromgrainin A in cerebellar cortex of mother’s rats (PND-21 M) and their 21-day old offspring (PND-21P). The chromogranin A immune reactivity appeared prominent in LPS groups compared with the other studied groups. In LPS-treated rats and their pups, the degree of chromogranin A immune-reactivity appears less intensive than that in LPS-group. The arrow heads point to the chromogranin A immune reactivity. (Stain: Chromogranin A antibody, Scale bar: 25 µm). Panels E&E1 showing the quantitative analysis of chromogranin A positively expressed cells of cerebellar cortex among the control and experimental groups of mother rats and their pups.

The cerebellar cortex sections from control and Zn-NPs supplemented maternal rats (Figure 11A–B) and their progeny (Figure 11A1-B1) exhibited negative expression of chromogranin A in the granular and basket cells, while Purkinje cells demonstrated minimal expression in mothers and negative expression in their offspring. In comparison to the control group, female rats given with LPS (Figure 11C) and their offspring (Figure 11C1) exhibited pronounced positive expression of chromogranin A protein. This expression was more concentrated in the three varieties of cerebellar cortex cells. Conversely, the cerebellar sections from mothers and their offspring exhibited a negative chromogranin A reaction subsequent to treatment with LPS, followed by treatment with Zn-NPs (Figure 11 D-D1). The quantitative analysis depicted in (Figure 11E, E1) indicates that the levels of chromogranin A-positive cerebellar cells were considerably elevated in LPS-treated mother rats and their offspring (3.896 for mothers and 3.375 for pups) compared to the control group (0.168 and 0.05, respectively). However, following treatment with Zn-NPs, the number of chromogranin A positive cells dramatically decreased but did not return to the normal levels observed in the control group (0.688 for mothers and 0.583 for pups).

### Synaptophysin immunohistochemical activity

**Fig. 12:**
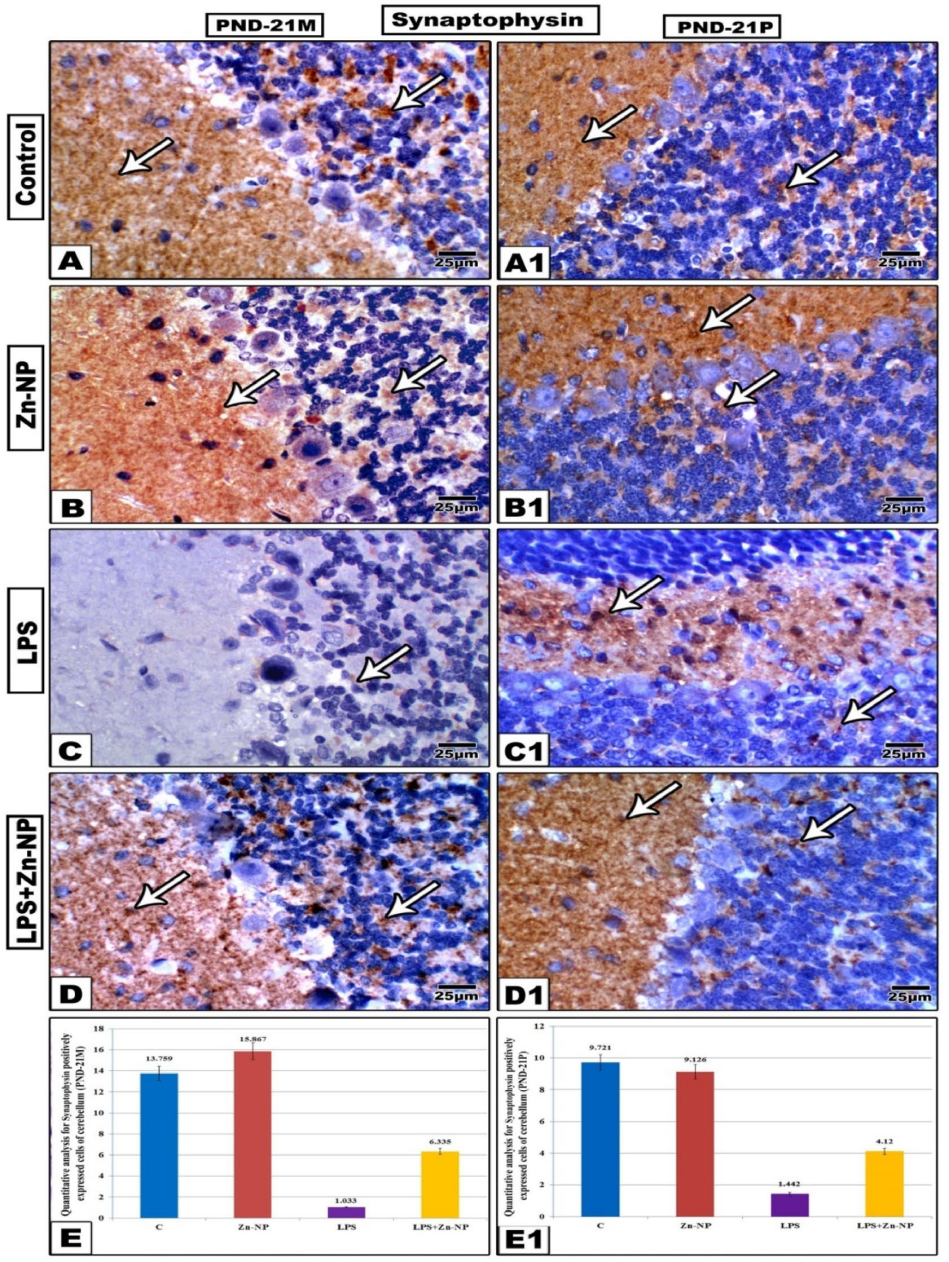
Immunohistochemical expression of synaptophysin in the cerebellar cortex of mother’s rats (PND-21 M) and their 21-day old offspring (PND-21P). Synaptophysin immune reactivity appeared less in LPS groups compared with the other studied groups. In LPS-treated rats and their pups, the degree of synaptophysin immune-reactivity appears more intensive than that in LPS-group. Arrow heads point to the synaptophysin immune reactivity. (Stain: synaptophysin antibody, Scale bar: 25 µm). Panels E & E1 showing the quantitative analysis of synaptophysin positively expressed cells of cerebellar cortex among the control and experimental groups of mother rats and their pups.

In control and Zn-NPs supplemented mother’s rat (Figure12A–B) and their offspring (Figure A1-B1), the cerebellar sections displayed strong positive expression for synaptophysin. This expression was more localized in the molecular layer^61^ than the granular cells while negative in Purkinje cells. However, in LPS-administered mother rats (Figure 12C) and their offspring (Figure 12C), the cerebellar sections appeared negatively or very weakly stained with synaptophysin antibody if compared with control. On the other hand, the LPS-administered rats followed by Zn-NPs supplementation, the cerebellar sections displayed moderate immune expression for synaptophysin (Figure 12D, D1). As shown in (Figure 12E, E1), the quantitative analysis of synaptophysin positively cerebellar expressed cells appeared significantly lower in LPS-administered mother rats and their offspring if compared with the control. The quantitative analysis of synaptophysin-positive cells in the cerebellar cortex (Figure 12E, E1) revealed that the mean numbers of positive cells in the control, Zn-NPs, LPS, and LPS+Zn-NPs groups were 13.76 ± 1.2, 15.86 ± 1.4, 1.03 ± 0.2, and 6.34 ± 0.8, respectively, in mother rats, and 9.21 ± 0.9, 9.13 ± 0.8, 1.44 ± 0.3, and 4.12 ± 0.5, respectively, in their pups.

### Neuron specific enolase (NSE) immunohistochemical activity

**Fig. 13:**
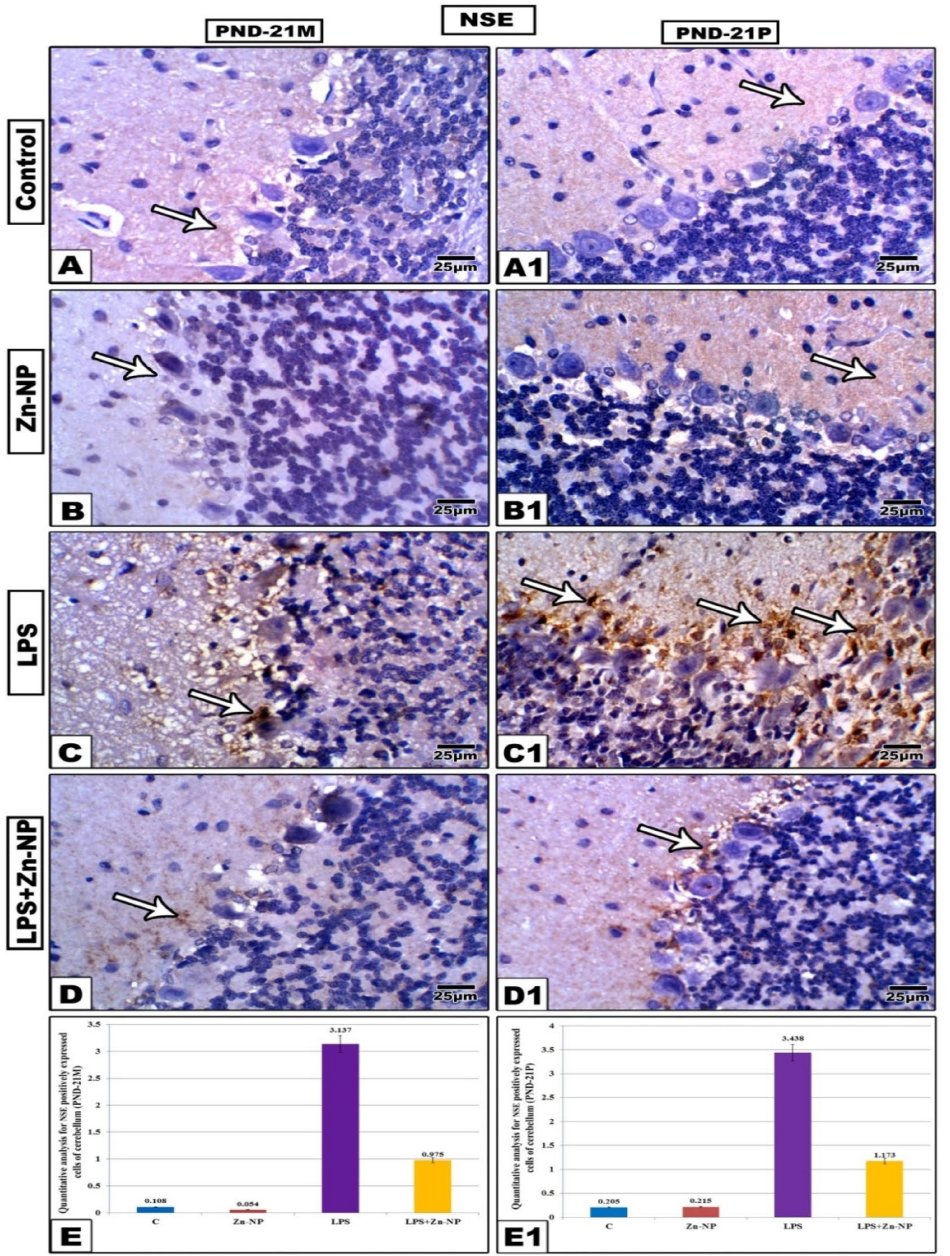
Representative neuron-specific enolase (NSE) immunostaining in the cerebellar cortex of mother’s rats (PND-21 M) and their 21-day old offspring (PND-21P). The NSE immune reactivity appeared significantly higher in LPS group in comparison with the other studied groups. In LPS-treated rats and their pups, the degree of NSE immune-reactivity appears less intensive than that in LPS-group. Arrow heads point to the NSE immune reactivity. (Stain: neuron-specific enolase antibody, Scale bar: 25 µm). Panels E&E1 showing the quantitative analysis of NSE positively expressed cells of cerebellar cortex among the control and experimental groups of mother rats and their pups.

In control and Zn-NPs supplemented female rats (Figure 13A, B) and their offspring (Figure 13A, A1), the cerebellar sections displayed very weak expression for neuron specific enolase (NSE) especially in the molecular and granular cell layers while Purkinje cells appeared negatively stained. However, in LPS-administered mother rats and their offspring, the three layers of cerebellar cortex appeared highly positively stained for NSE if compared with the control (Figure 13C, C1). In LPS-administered rat dams and post-supplemented with Zn-NPs and PND 21 offspring, the cerebellar section displayed very weak immune reactivity for NSE which appeared more or less like the control (Figure 13D, D1). As shown in (Figure 13E, E1), the quantitative analysis of NSE positively expressed cells of cerebellar cortex appeared significantly higher in LPS-administered female rats and their offspring if compared with their control. The positively NSE expressed cells among the control, Zn-NPs, LPS, and LPS+ Zn-NPs groups of mothers were 0.108, 0.054, 3.137, and 0.975 respectively, while in their pups were 0.205, 0.215, 3.438, and 1.73 respectively.

## Flow cytometric analysis of P53, TNF-α, and annexin-V

### Changes in P53

**Fig. 14:**
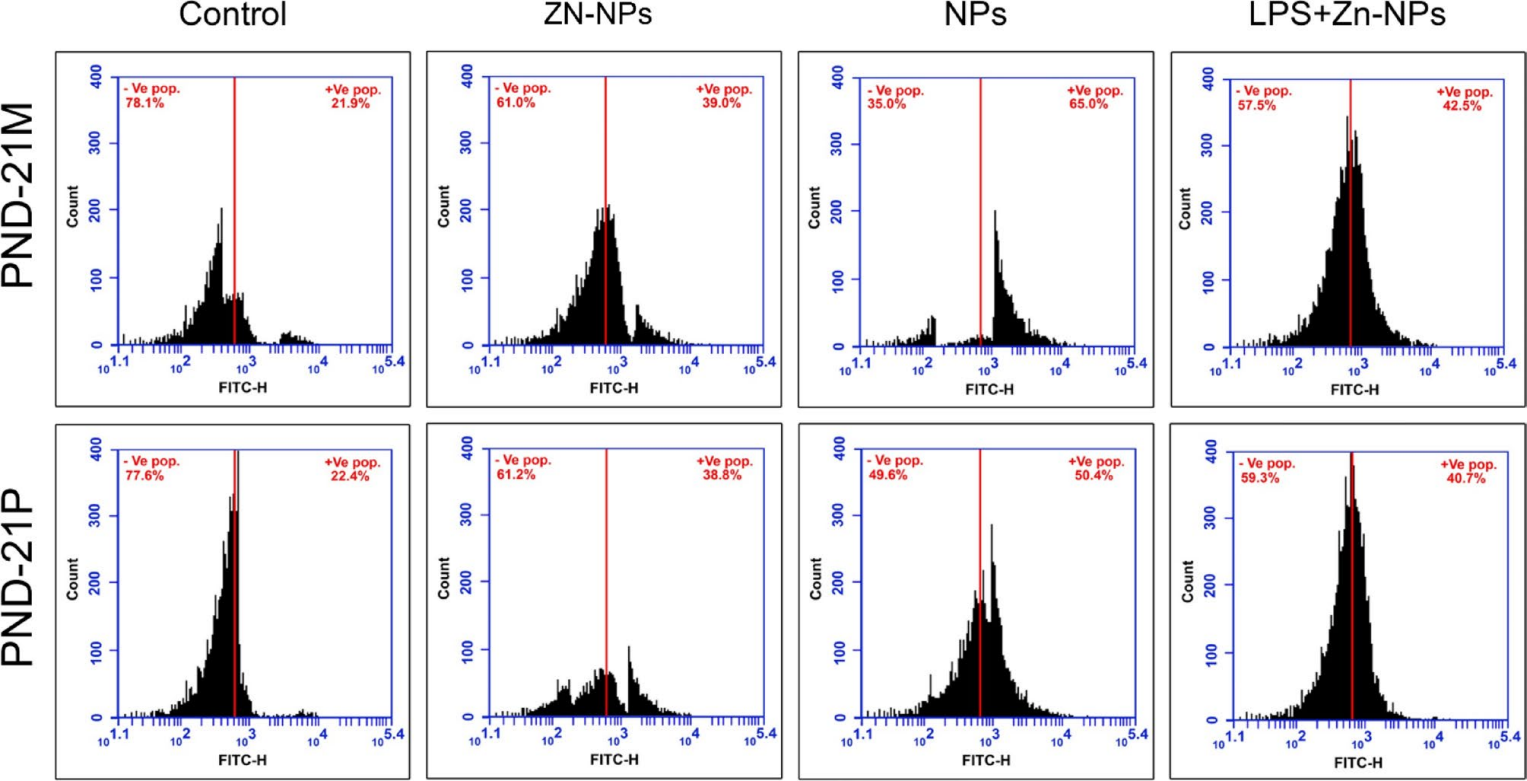
A flow cytometric chart illustrating the mean % of P53 positively expressed cells in the cerebellar tissues of mother rats (PND-21 M) and their pups at PND 21(PND-21P) using FITC-H Scan analysis. The highest % values of P53 appears in the LPS-administered mother’s rats and their offspring. The LPS + Zn-NPs treated rats and their pups, the percentage of P53 values appear lower than the LPS-administered group.

Figure 14 illustrates the average percentage of P53-positive cells in the cerebellar cortex of maternal rats and their offspring. In LPS-treated mother rats and their offspring, the proportion of P53-positive cells was markedly elevated (65% in mothers and 50.4% in pups) relative to the control group (21.9% in moms and 22.4% in offspring). Nonetheless, following treatment with Zn-NPs, the mean percentage of P53-positive cells drastically decreased in comparison to the LPS group, yet remained much higher than the control (42.5% for moms and 40.7% for pups). Gestational exposure of pregnant rats to bacterial LPS is associated with the induction of cerebellar cell apoptosis via increased P53 activity in the mothers compared to their offspring (65% vs. 50.4%).

### Changes in TNF-α

**Fig. 15:**
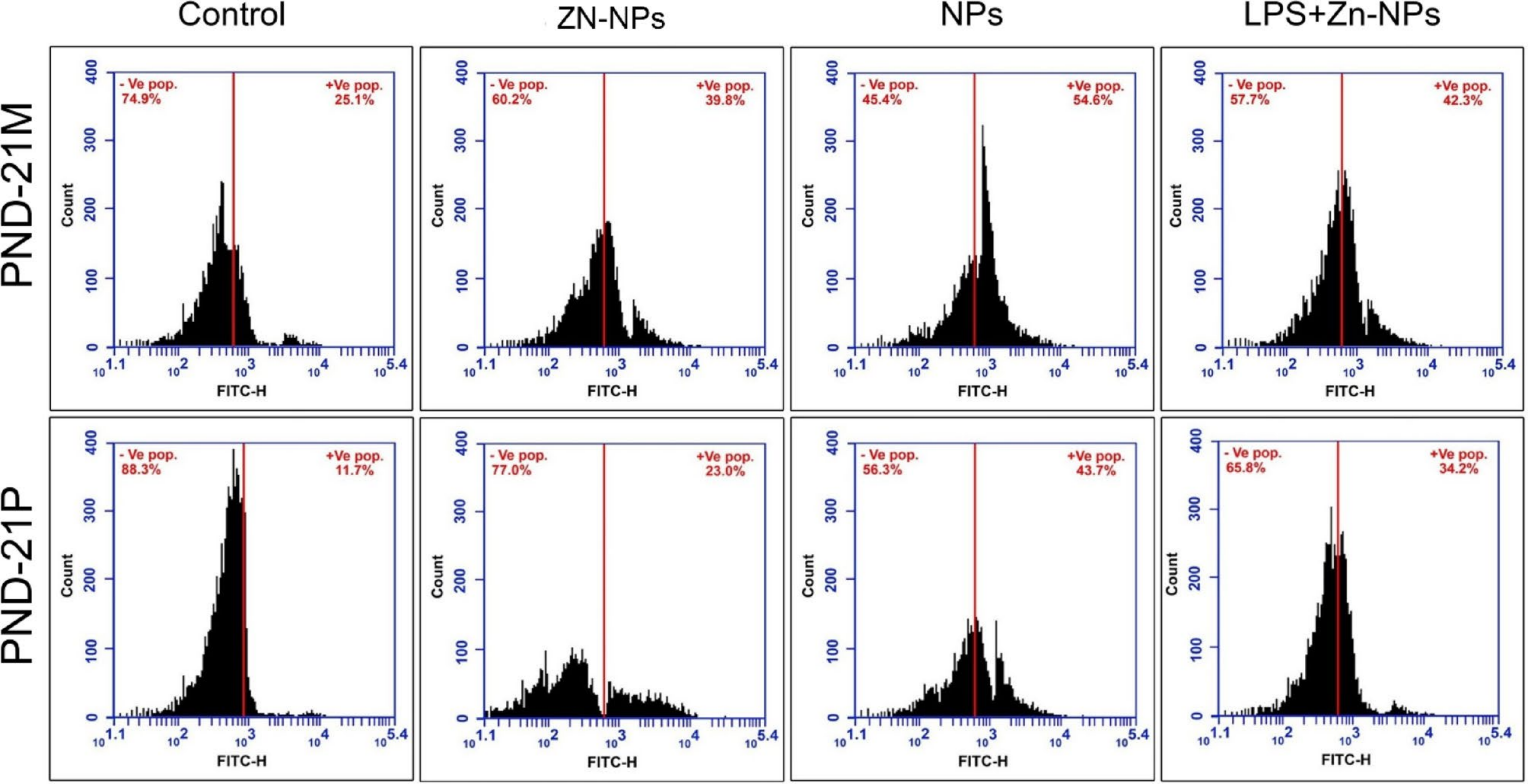
A flow cytometric chart illustrating the mean % of TNF-α positively expressed cell in the cerebellar tissues of mother rats (PND-21 M) and their pups at PND 21(PND-21P) using FITC-H Scan analysis. The highest % values of TNF-α appear in the LPS-administered mother’s rats and their offspring. The LPS + Zn-NPs treated rats and their offspring, the percentage of TNF-α values appear lower than the LPS-administered group.

As shown in figure 15, the mean percentages value of TNF-α positively expressed cells from the cerebellar cortex of LPS-administered mother rats and their offspring appeared significantly higher (54.6% for mothers and 43.7% for pups) than the control (25.1% for mothers and 11.7% for pups). However, in LPS-administered mothers followed by treatment with Zn-NPs, the mean percentage value of TNF-α positively expressed cells were markedly decreased (42.3% for mothers and 34.2% for pups) if compared with LPS-group alone but still significantly higher than the control. Exposure of pregnant rats to bacterial LPS during gestation is implicated in induction of inflammation in the cerebellar cells through up-regulation of TNF-α activity in mothers higher than their pups (54.6% Vs43.7%).

### Changes in annexin-v (apoptotic and necrotic marker)

**Fig. 16:**
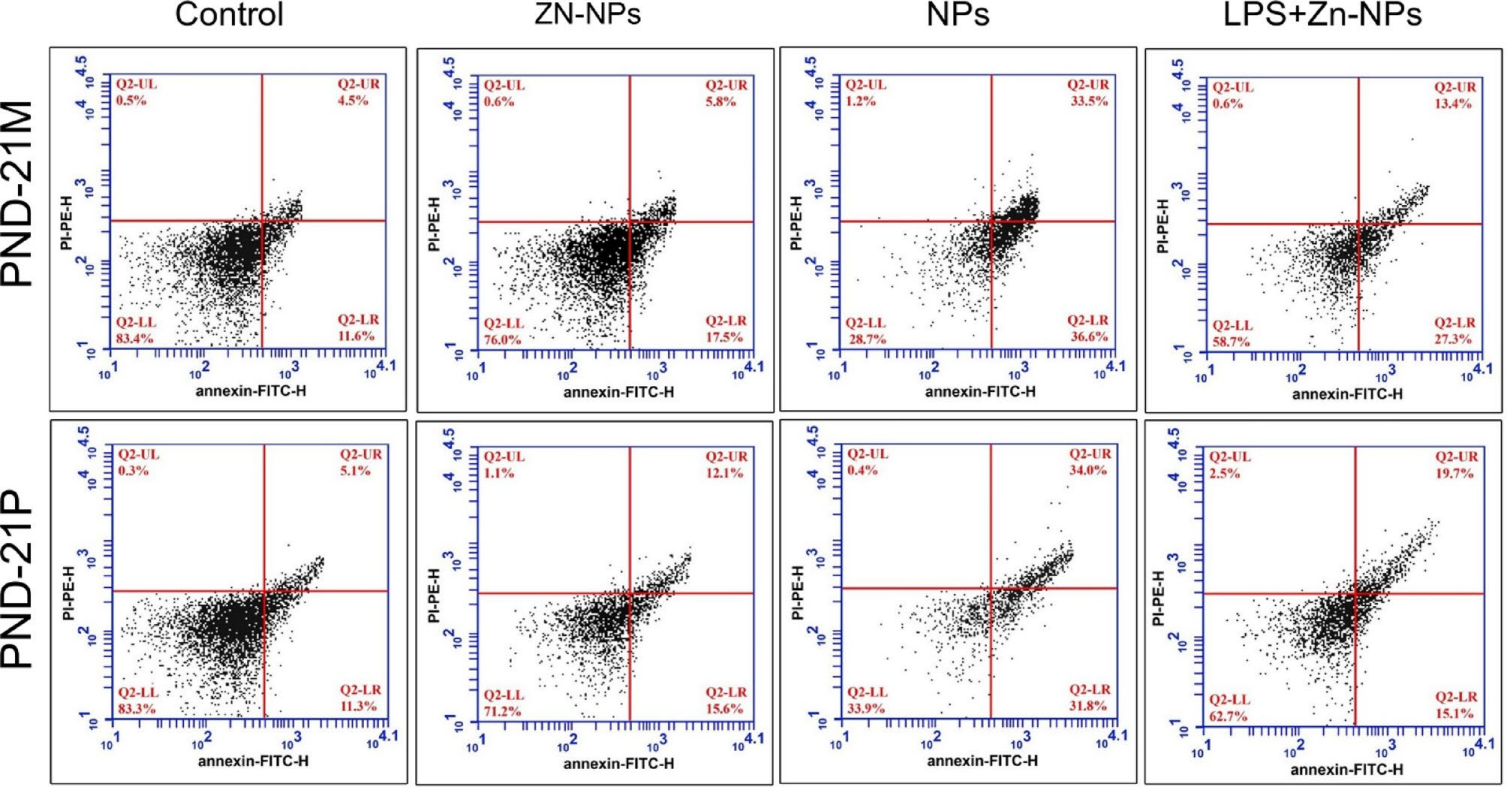
A flow cytometric chart illustrating the mean % of apoptosis and necrosis by PI-PE-H Scan analysis via Annexin V- FITC-H in the cerebellar tissues among control and the different studied groups of mother rats (PND-21 M) and their pups at PND 21(PND-21P). Cells in the lower left quadrant indicate the % of Annexin-negative/PI-negative (viable cells). Cells in the lower right quadrant indicate the % of Annexin-positive/PI-negative (early apoptotic cells). Cells in upper left quadrant indicate the % of Annexin-negative/PI-positive (necrotic cells). Cells in upper right quadrant indicate the % of Annexin-positive/ PI-positive^90^. The percentage of early and late apoptosis appears significantly higher in LPS-administered rats and their offspring while after treatment with Zn-NPs- treated rats and their offspring, these percentages appear lower than the LPS-administered group.

In control and Zn-NPs supplemented maternal rats, the mean percentage of viable cerebellar cells was 83.4% and 76.0% (lower left quarter panel), the mean percentage of late apoptotic cells was 4.5% and 5.8% (upper right quarter panel), the mean percentage of early apoptotic cells was 11.6% and 14.5% (lower right quarter panel), and the percentage of necrotic cells was 0.5% and 0.6% (upper left quarter panel), respectively (Figure 16A, B). Conversely, the cerebellar cells from LPS-treated maternal rats exhibited a markedly significant reduction in the mean percentage of viable cells (28.7%) and a substantial increase in late apoptotic cells (33.5%), early apoptotic cells (36.6%), and necrotic cells^88^ when compared to the control (Figure 16C). Following treatment with Zn-NPs, the percentage of viable cells (58.7%) significantly increased, while the proportions of late apoptotic cells (13.4%), early apoptotic cells (27.3%), and necrotic cells (0.6%) significantly decreased in comparison to the LPS-administered group alone, although they did not attain the standard value observed in the control group (Figure 16D).

In the control and Zn-NPs maternally supplemented offspring, the mean percentage of viable cerebellar cells was 83.3% and 71.2%, respectively (lower left quarter panel). The mean percentage of late apoptotic cells was 5.1% and 12.1% (upper right quarter panel), while the mean percentage of early apoptotic cells was 11.3% and 15.6% (lower right quarter panel). The percentage of necrotic cells was 0.3% and 1.1% (upper left quarter panel), respectively (Figure 16A1 and B1). Conversely, cerebellar cells from offspring maternally exposed to bacterial LPS exhibited a markedly significant reduction in the mean percentage of viable cells (33.9%) and a substantial increase in the percentages of late apoptotic cells (34.0%) and early apoptotic cells (31.8%), while the change in necrotic cells remained non-significant (0.4%) when compared to the control (Figure 16C1). The progeny of LPS-treated maternal rats subsequently supplemented with Zn-NPs exhibited a statistically significant increase in the percentage of viable cells (62.7%), alongside a notable decrease in late apoptotic cells (19.7%) and early apoptotic cells (15.1%), as well as a significant increase in necrotic cells^89^ when compared to the LPS-induced group; however, these values did not attain the standard set by the control group (Figure 16 D1).

## Discussion

Lipopolysaccharide (LPS) is frequently utilized as a model for bacterial pathogens^13,91^. Maternal exposure to LPS at various gestational stages correlated with negative pregnancy outcomes in mouse models^22,23,89,92,92,93^. Zinc nanoparticles are innovative materials exhibiting significant biological properties and minimal toxicity, demonstrating a pronounced ability to overcome various challenges associated with effective cellular and molecular targeting in numerous disorders^94–96^. The primary objective of this work was to evaluate the possible function of Zn-NPs in mitigating bacterial LPS-induced cerebellar damage in pregnant rats and their offspring.

The results indicated a substantial reduction in body weight among LPS-treated mothers (at GD16 and PND-21) and their offspring (at PND1, 7, 14, and 21) relative to the control group. However, following treatment with Zn-NPs, body weight significantly increased compared to the LPS group, yet remained considerably lower than that of the control group. The results align with the findings of Wang et al.^25^, which indicated that exposure to LPS during mid-gestation significantly reduces the body weight of pregnant mice and their offspring. The diminished body weight of LPS-exposed pregnant rats may result from reduced food consumption, while LPS may contribute to appetite suppression; however, the reduced body weight of their offspring is primarily linked to intrauterine oxidative stress induced by LPS on the placenta, in addition to lactation feeding practices^13,97^. The restoration of body weight in LPS-treated pregnant rats and their offspring to near-normal levels following Zn-NP supplementation aligns with the findings of Pei et al.^98^, who indicated that low doses of Zn-NPs significantly contribute to growth performance by enhancing food intake and preserving intestinal flora. Subsequent research on developing animals demonstrated that Zn-NPs enhance food digestion and absorption, hence reducing weight disorders^99^.

Dopamine is a prevalent neurotransmitter in the central nervous system and is essential for numerous cognitive activities^100^. Serotonin levels have been associated with learning and memory consolidation^101^. This experiment revealed that serum levels of dopamine (DA) and serotonin^84^ in LPS-administered female rats and their offspring were significantly lower than those in the control group. Following treatment with zinc nanoparticles^102^, the levels of DA and 5-HT increased dramatically, however remained markedly lower than the control levels. This indicates that LPS can interfere with neurotransmitter action, but zinc nanoparticles may enhance neurotransmitter levels in the CNS. Bacterial LPS was observed to enhance serotonin metabolism in rats by inhibiting serotonin transporters^103^. LPS was shown to impair dopamine-producing neurons via the activation of microglial cells, leading to dopamine depletion^104^. Goma et al.^57^shown that Zn-NPs can sustain circulating dopamine levels, as zinc regulates the dopamine transporter by directly interacting with the transporter protein, serving as a powerful non-competitive inhibitor of substrate translocation. Furthermore, it has been revealed that zinc is a vital vitamin that may alleviate symptoms of anxiety and depression in certain individuals by regulating serotonin levels^105^.

Reactive oxygen species (ROS) are generated in surplus relative to the rate at which antioxidants can eliminate them, a condition referred to as oxidative stress^106^. Exposure to LPS has been found to activate macrophages, resulting in excessive generation of ROS linked with significant inflammation^107–109^. The present study indicates that LPS exposure during gestation leads to a decrease in cerebellar antioxidants like SOD, CAT, and GSH in both mothers and their offspring, while levels of lipid peroxidation indicators, including MDA and NO, were elevated relative to the control group. The reduction of endogenous enzymatic and non-enzymatic antioxidants has been seen in the brain**; **^110,111^ as well as in the ovaries and placenta﻿ ^13^ of LPS-induced pregnant rats. Additional investigations indicated that LPS can provoke lipid peroxidation both in vitro^112,113^ and in vivo^114^ in animal models. This indicates that bacterial LPS is involved in the production of oxidative stress in cerebellar tissues.

Remarkably, Zn-NPs significantly regulated the cerebellar antioxidants and oxidative stress towards normalcy in LPS-administered rats and their offspring. This indicates the potential of zinc nanoparticles in mitigating oxidative stress. Zinc is essential for the manufacture of critical antioxidant enzymes, and its shortage disrupts this process, leading to heightened oxidative stress^115^. Zinc serves as a cofactor for numerous enzymes and is vital for various biological activities, including antioxidant defense mechanisms. The data indicates that Zn-NPs exhibit antioxidant capabilities. These results corroborate recent findings regarding the modulation of the antioxidant response of Zn-NPs in brain tissues^59,116^. Zinc oxide nanoparticles (ZnONPs) have demonstrated protective effects on the brain, counteracting cognitive decline and anxiety related to aging while alleviating oxidative damage^117^. Zinc is vital for the activity of antioxidant enzymes such as superoxide dismutase (SOD) and is recognized for safeguarding sulfhydryl groups by displacing other metals from catalytic sites. Consequently, Zn-NPs can maintain the integrity of cell membranes by protecting them from oxidative damage, enhancing antioxidant levels, and diminishing free radicals^13,118^.

Prior research has demonstrated a variance in lipid profile levels among patients with various infections (bacterial, viral, and parasitic). For example, TC, LDL-C, and HDL-C levels are diminished, although plasma triglyceride levels are increased or remain within normal limits after various infections^119–121^. Our findings indicated that LPS exposure during gestation elevated serum levels of total cholesterol, triglycerides, and LDL in both dams and their offspring, while significantly reducing HDL relative to the control group. This indicates that bacterial LPS may influence hepatic fat metabolism by degrading liver lipids into free fatty acids and cholesterol. Although LDL-C levels were reduced, the quantity of small dense LDL was observed to increase during infections^122,123^. Epidemiologic research indicates that low levels of cholesterol, LDL-C, and HDL elevate the risk of infection^124,125^. The divergence in our results from the prior findings may be ascribed to the dosage of LPS and the length of the infection. Future studies with larger sample sizes specifically powered to detect sex-dependent effects would be valuable to determine whether male and female offspring differ in their susceptibility to LPS-induced neurotoxicity or their response to Zn-NP treatment.

Research on animals has demonstrated that zinc is crucial for fatty acid and glucose metabolism^126^. Both vivo and in vitro experiments demonstrated that zinc supplementation mitigates lipid and glucose metabolic problems caused by a high-fat diet^127^. In this study, the feeding of pregnant rats with Zn-NPs from gestational day 15 till weaning markedly enhanced the lipid profiles in rats given with LPS. Numerous human investigations have shown that Zinc supplementation decreases total cholesterol, LDL cholesterol, and triglycerides, while simultaneously elevating HDL cholesterol levels^128,129^. A comparable study indicated that zinc supplementation (100mg/day) may positively affect the lipid profile in individuals with type-2 diabetes by normalizing TC, low-density lipoprotein cholesterol (LDL-C), (HDL-C, and TG^130^. Zinc supplementation can enhance lipid profiles via multiple processes, such as facilitating lipolysis, suppressing lipogenesis, and modulating the activity of certain enzymes and proteins associated with lipid metabolism. Zinc regulates adipokines, hormones that influence fat storage and metabolism^131,132^.

The present study demonstrates that Zn-NPs effectively alleviated the cerebellar histopathological alterations induced by LPS, including pyknotic or lysed Purkinje cells, atrophied and vacuolated granular cells, as well as ultrastructural features such as pyknotic hyperchromatic nuclei, atrophied mitochondria, dilated rough endoplasmic reticulum, damaged nuclei, and irregular nuclear membranes. These findings demonstrate the anti-inflammatory and therapeutic effects of zinc against harmful histopathological damage caused by LPS. The results obtained align with prior research regarding the impact of zinc oxide nanoparticles on brain tissue structure^25,133,134^. While the method by which zinc mitigates cerebellar neural cell damage remains ambiguous, prior studies indicate that zinc may reduce cellular damage chiefly through its antioxidant and membrane-stabilizing attributes. It also plays a vital role in safeguarding cells from oxidative stress, which may result in many forms of damage, including DNA damage and apoptosis^60,135^. Moreover, zinc can avert cellular harm by stimulating the expression of metallothioneins. These metal-binding, cysteine-rich proteins are crucial for regulating zinc-related cellular homeostasis and function as effective electrophilic scavengers and cytoprotective agents^136^.

Chromogranin A is an acidic protein that constitutes a significant component of the secretory granules in many endocrine and neuroendocrine cells^137^. Increased concentrations of Chromogranin A correlate with neurotoxicity and neurodegenerative mechanisms, primarily via the activation of microglia, the brain’s intrinsic immune cells^138^. The present study revealed that LPS-treated female rats and their offspring had pronounced chromogranin A protein expression in the cerebellar cortex cells relative to the control group; however, following treatment with Zn-NPs, the cerebellar tissues displayed negative staining for this protein. An increase in serum Chromogranin A in a distinct kind of sepsis caused by gram-negative bacteria has been documented, corroborating our findings^139^. The observed negative Chromogranin A expression following treatment with Zn-NPs in this study may be ascribed to the antioxidant properties of zinc in countering oxidative stress generated by LPS. The mechanism via which zinc reduces Chromogranin A expression remains ambiguous; yet, a prior work by Li et al.^40^confirmed that low dosages of zinc can mitigate neurotoxicity in several neurological disorders.

Synaptophysin is a prevalent integral membrane glycoprotein found in the presynaptic vesicles of neurons. Robust immunohistochemical expression of synaptophysin signifies well-synapsed neurons, whereas diminished or absent expression indicates degenerative neurons^140^. In our work, the cerebellar cortex of mother rats treated LPS exhibited neurons with negative synaptophysin staining, whilst their pups displayed mild staining in comparison to the control; nevertheless, treatment with Zn-NPs resulted in significantly enhanced synaptophysin immunoreactivity. This indicates that LPS is involved in neuroinflammation and degeneration, whereas zinc is essential for the synaptic capacity of cerebellar neurons through the creation of synaptophysin. Zinc is essential for preserving neuronal integrity and significantly contributes to synapse development and function via its interaction with synaptophysin. Zinc is prevalent in the brain and participates in numerous neural processes^141^.

Neuron-specific enolase (NSE) is an isomer of the enzyme enolase, located in neurons and neuroendocrine cells. Upon neuronal injury, it may be released into the cerebrospinal fluid^142^ and can be quantified to assess specific medical disorders, such as brain injury and some cancers^143^. The current findings demonstrate elevated NSE expression in the cerebellar molecular layer cells of LPS-treated mother rats and their offspring compared to the control; however, following treatment with Zn-NPs, this expression significantly decreased. This verifies the role of LPS in inducing cerebellar injury, while Zn-NPs alleviate this injury. NSE levels were observed to rise in correlation with the severity of bacterial infections, TB, and inflammation^144–146^. Prior research indicated that zinc plays several essential roles in the neurological system. It serves as a synaptic neuromodulatory signal, an intracellular initiator of several signal transduction pathways, and a structural and catalytic element of essential proteins^147^.

The P53 protein is a DNA-binding entity that plays a role in transcription control^148^. The overexpression of p53 correlates with lesions exhibiting high-risk characteristics, including an elevated apoptotic rate. Conversely, diminished expression of P53 protein promotes aberrant cell division and tumor growth^149^. TNF-α is recognized as a principal regulator of inflammatory responses and is implicated in the pathophysiology of various inflammatory and autoimmune illnesses^150–152^. FITC Annexin V quantitatively assesses the proportion of cells in a population that are undergoing apoptosis and necrosis. In flow cytometry, annexin V is frequently employed to identify apoptotic cells due to its capacity to bind to phosphatidylserine, a hallmark of apoptosis when located on the outer membrane of cells^153^.

The flow cytometric analysis conducted in this study, utilizing FITC P53, TNF-α, and annexin-V to quantitatively assess the percentage of cells in cerebellar tissues undergoing apoptosis, revealed a significant increase in both LPS-treated mother rats and their offspring compared to the control group. Nonetheless, following treatment with Zn-NPs from GD15 to weaning, this percentage drastically decreased, albeit remaining considerably greater than the control group. This indicates the detrimental effects of bacterial LPS in causing cerebellar cell damage through the activation of apoptosis and necrosis, while Zn-NPs may play a role in modulating LPS-induced apoptosis. In contrast to our findings, Aschtgen et al. **2022** found diminished P53 activation and tumor growth following LPS exposure. This conclusion may be ascribed to the dosage and duration of exposure to LPS. Karahashi et al. **2009** demonstrated that LPS can indirectly provoke inflammation and cell death through the release of TNF-α in macrophages or endothelial cells. Apoptosis of neuronal cells in dental pulp infected with bacterial lipopolysaccharides and elevated production of TNF-α. According to Sampoerno et al.^154^, LPS was observed to cause cognitive impairment and neuroinflammation in C57BL/6J mice through the elevation of pro-inflammatory cytokines such as TNFα^155^. LPS was identified as a mediator of inflammatory effects on cell cycle re-entry and apoptosis in neuronal cells, consistent with our findings on the overexpression of annexin-V^156^. Annexin V was discovered to bind to Gram-negative bacteria through the lipid A domain of lipopolysaccharides, resulting in apoptosis and necrosis^157^.

The data acquired about the anti-apoptotic properties of zinc in this investigation align with the findings of Noshy et al.^59^, who demonstrated that Zn-NPs can mitigate oxidative stress and apoptosis generated by silver nanoparticles in the brains of male rats. Zinc was discovered to facilitate the neuronal activity-dependent anti-apoptotic action by inhibiting caspase-3 activity, a pro-apoptotic signal^158^. A separate study indicated that zinc can influence apoptosis by regulating P53 expression^159^. The precise method via which Zn-NPs reduce cerebellar cell death remains unidentified. The protective action of zinc is attributed to its suppression of a Ca2+ and Mg2+-dependent endonuclease, which prevents DNA fragmentation, a definitive step and characteristic of apoptosis^160^. Furthermore, prior research has validated the antioxidative and anti-apoptotic properties of Zn-NPs, as well as their capacity to diminish ROS production and oxidative stress in various rat organs^103^.

## Conclusion

Our findings indicated that exposure to bacterial LPS during gestation is linked to cerebellar neurotoxicity in maternal rats and their offspring, as demonstrated by the induction of oxidative stress, histopathological alterations, and apoptosis, along with major changes in neurotransmitter levels and lipid profiles. Zinc nanoparticles effectively mitigated cerebellar neurotoxicity generated by LPS in both mothers and their offspring by modulating antioxidants, apoptotic markers, lipid profiles, and neurotransmitters. This was accompanied by the return of cerebellar histogenesis to near normalcy.

## Data Availability

The datasets used and/or analyzed during the current study are available from the corresponding author on reasonable request.
